# Ensemble learning for poor prognosis predictions: a case study on SARS-CoV2

**DOI:** 10.1093/jamia/ocaa295

**Published:** 2020-11-13

**Authors:** Honghan Wu, Huayu Zhang, Andreas Karwath, Zina Ibrahim, Ting Shi, Xin Zhang, Kun Wang, Jiaxing Sun, Kevin Dhaliwal, Daniel Bean, Victor Roth Cardoso, Kezhi Li, James T Teo, Amitava Banerjee, Fang Gao-Smith, Tony Whitehouse, Tonny Veenith, Georgios V Gkoutos, Xiaodong Wu, Richard Dobson, Bruce Guthrie

**Affiliations:** 1 Institute of Health Informatics, University College London, London, U.K; 2 Health Data Research UK, University College London, London, U.K; 3 Centre for Medical Informatics, Usher Institute, University of Edinburgh, Edinburgh, U.K; 4 Institute of Cancer and Genomic Sciences, University of Birmingham, Birmingham, U.K; 5 Department of Biostatistics and Health Informatics, Institute of Psychiatry, Psychology and Neuroscience, King’s College London, London, U.K; 6 Centre for Global Health, Usher Institute, University of Edinburgh, Edinburgh, U.K; 7 Department of Pulmonary and Critical Care Medicine, People’s Liberation Army Joint Logistic Support Force 920th Hospital, Yunnan, China; 8 Department of Pulmonary and Critical Care Medicine, Shanghai East Hospital, Tongji University, Shanghai, China; 9 Centre for Inflammation Research, Queens Medical Research Institute, University of Edinburgh, U.K. Department of Respiratory Medicine, Royal Infirmary of Edinburgh, NHS Lothian; 10 Department of Stroke and Neurology, King’s College Hospital NHS Foundation Trust, London, U.K; 11 Department of Intensive Care Medicine, Queen Elizabeth Hospital Birmingham, Edgbaston, Birmingham, U.K; 12 Birmingham Acute Care Research, University of Birmingham, Birmingham, U.K; 13 Institute of Translational Medicine, University Hospitals Birmingham NHS Foundation Trust, Birmingham, United Kingdom; 14 Health Data Research UK, Midlands; 15 Department of Pulmonary and Critical Care Medicine, Taikang Tongji Hospital, Wuhan, China; 16 Centre for Population Health Sciences, Usher Institute, University of Edinburgh, Edinburgh, U.K

## Abstract

**Objective:**

Risk prediction models are widely used to inform evidence-based clinical decision making. However, few models developed from single cohorts can perform consistently well at population level where diverse prognoses exist (such as the SARS-CoV2 pandemic). This study aims at tackling this challenge by synergising prediction models from the literature using ensemble learning.

**Materials and Methods:**

In this study we selected and reimplemented seven prediction models for COVID-19, which were derived from diverse cohorts and used different implementation techniques. A novel ensemble learning framework was proposed to synergise them for realising personalised predictions for individual patients. Four diverse international cohorts (2 from the UK and 2 from China; total N=5,394) were used to validate all eight models on discrimination, calibration and clinical usefulness.

**Results:**

Results showed that individual prediction models could perform well on some cohorts while poorly on others. Conversely, the ensemble model achieved the best performances consistently on all metrics quantifying discrimination, calibration and clinical usefulness. Performance disparities were observed in cohorts from the two countries: all models achieved better performances on the China cohorts.

**Discussion:**

When individual models were learned from complementary cohorts, the synergised model will have the potential to achieve synergised performances. Results indicate that blood parameters and physiological measurements might have better predictive powers when collected early, which remains to be confirmed by further studies.

**Conclusions:**

Combining a diverse set of individual prediction models, ensemble method can synergise a robust and well-performing model by choosing the most competent ones for individual patients.

https://mc.manuscriptcentral.com/jamia

## Conclusions

Combining a diverse set of individual prediction models, ensemble method can synergise a robust and well-performing model by choosing the most competent ones for individual patients.

https://mc.manuscriptcentral.com/jamia

## Introduction

Risk prediction models are widely used in clinical practice to inform decision making.[1–3] Good models cannot only improve health service efficiencies but also predict deterioration[4] in a proactive manner [5], with a great potential to improve outcomes and save lives. Such evidence based decision making supports are particularly important in an epidemic or pandemic outbreak, not only for informing the treatments/managements of those infected but also for optimising healthcare services to minimise indirect effects to most vulnerable service users. For example, the recent SARS-CoV2 has caused substantial excess mortality [6,7] at least partly due to an indirect effect upon healthcare systems leading to a loss of capacity to provide elective and emergency care within the “golden window” of opportunity [7–9]. To mitigate excess mortality, more targeted inpatient care in future waves could be informed by (a) better risk prediction and (b) insights from international COVID-19 (we use SARS-CoV2 and COVID-19 interchangeably) datasets and experience to validate models and learn from different countries’ responses.

There have been numerous prediction models developed for COVID-19, [10–14] but most were derived in small datasets, had low methodological quality and are unvalidated.[13] In addition, models learned from single cohorts (even from several centres) might not have the predictive power to achieve good performance in situations where a disease spreads to the whole population, leading to greatly diverse prognoses. In this study, we reproduced various prediction models with reasonable quality and synergised them using ensemble learning [15] to assess their *collective* ability to accurately discriminate mild and severe patients in a diverse set of four patient cohorts from the UK and China with varying patterns of disease severity (Figure 1a). In particular, China and the UK had very different approaches to hospital admission for COVID-19. In Wuhan, admission was routine with patients triaged to low intensity (Fangcang hospitals[16]) or higher dependency (designated hospitals) settings, whereas in the UK, admission of patients with more severe disease or at perceived higher risk of severe disease was prioritised. These differences enabled us to assess model performance in different settings. For outcomes https://mc.manuscriptcentral.com/jamia specifically, we primarily focused on poor prognosis defined by either death or intensive care unit (ICU) stay.

## Materials and Methods

Figure 2 depicts the architecture of this work - synergising individual models from the literature for preventing excess mortality. For prediction models (Figure 1b), seven models (Dong, [10] Shi, [17] Gong, [18] Lu, [19] Yan, [20] Xie, [21] and Levy [22]) were chosen with different model types using diverse sets of predictors. Derivation cohorts were diverse, originating from six regions in two countries, with median ages ranging from 44-65 years, and with mortality varying between 7-52%. Such diversity provides leverage for synergising insights from these derivation cohorts to obtain a collective, and hopefully improved predictive power.

To synergise models derived from multinational datasets, we used ensemble learning, [15,23] a machine learning methodology which is particularly effective when single models perform well at certain subsets of the whole data samples but none of them can achieve good overall performances. The rationale is to partition the data samples into groups and choose the most suitable model(s) for particular groups (e.g. to give more weights to models derived from older populations with more severe cases for a 78-year-old patient with lymphocyte count of 0.7) so that the optimal overall prediction result can be achieved. Figure 1c shows a synthetic and schematic illustration of such a situation. In (conventional) ensemble learning scenarios, weak predictors are usually trained on subsets of the same dataset. The key difference of this work is that the weak predictors were not trained locally on one particular dataset but selected from the literature, i.e. learned from external datasets (which the ensemble model does not have access to) and re-implemented for aggregation.

The aggregation approaches used in this study do not belong to the stacking method (also called stacked generalization [24]) that is to learn a new model using inputs from individual classifiers. Instead, they are inspired by bagging predictors [25] - aggregating results in a data-independence manner.

https://mc.manuscriptcentral.com/jamia

## Validation and analytics cohorts

The first Wuhan cohort (Wuhan01) consisted of 2,869 adults with COVID-19 confirmed by RT-PCR admitted to one of two hospitals in Wuhan, China (Wuhan Sixth Hospital and Taikang Tongji Hospital), admitted between 01/02/2020 and 23/02/2020, and who died or were discharged on or before 29/03/2020. The second Wuhan cohort (Wuhan02) consisted of 357 adults with COVID-19 from Tongji Hospital, data of which was collected between 10/01/2020 and 04/03/2020. The first UK cohort (KCH) consisted of 1,475 adults (≥18 years old) hospitalized with COVID-19 in King's College Hospital NHS Foundation Trust (London, United Kingdom) between 01/03/2020 and 02/04/2020, who have been followed up until 08/04/2020. The second UK cohort (UHB) consist of 693 adults (≥18 years old) hospitalized with COVID-19 at the Queen Elizabeth Hospital (part of the University Hospital Trust Birmingham, United Kingdom) between 14/03/2020 and 13/04/2020, who have been followed up to 19/04/2020. Mortality rates of Wuhan01, Wuhan02, KCH and UHB are 2.4%, 45.7%, 26.9% and 19.0% respectively. The large difference in mortality between two Wuhan cohorts was possibly because: (a) Wuhan02 admitted more severe cases under Wuhan city-wide coordination [20]; (b) the two were followed up in different periods related to the surge (Figure 1a.2). Table 1 gives the baseline for comparing poor-prognosis/died and not-poor-prognosis/did-not-die subgroups of all 4 cohorts. All cohorts were retrospective and extracted from Electronic Health Records for this study. Demographics and baselines of all four validation cohorts are described in detail in supplementary material (Table S2-S5).

## Prediction model selection and re-implementation

In May 2020, we conducted a literature search for COVID-19 poor prognosis models. The search and selection process are described with details in supplementary material Figure S1. Briefly, for prediction models (Figure 1b), we selected COVID-19 prognosis (either death or severity) models that were (a) reproducible (implementable models with all parameters reported); (b) using predictors that are readily available at community triage at large scale ( i.e.

https://mc.manuscriptcentral.com/jamia demographics, underlying conditions, blood tests, and vital signs); (c) with sufficient information describing the derivation cohort including cohort size, interquartile range (IQR) of age, country/region, follow-up period and mortality/poor-prognosis ratios. Table 2 describes information of the seven models including the outcomes, computational methods, information of derivation cohorts (size, region/country, mortality rate, follow-up period etc).

We re-implemented these seven prediction models by extracting all parameters from their published/preprint manuscripts or public-facing websites. 5 different models are implemented including decision tree, logistic regression, nomogram, scoring, and NOCOS (a customised transparent model). We also extracted derivation cohort size, follow-up periods, and distributions of numeric predictors (bloods and vitals). Supplementary Table S1 shows predictors used by each prediction model and also gives the numeric variable distributions of their derivation cohorts. Figure 1b illustrates the timeline of the follow-up periods of all models' derivation cohorts.

## Competence assessment framework for model selection

The key to obtaining an effective ensemble model is a good aggregation mechanism that can choose the best performing model(s) for individual patients so that an overall optimal classification could be achieved. Stacking methods (learning a model from individual classifiers) usually produce better ensembles than bagging (majority vote or weighted majority vote).[23] However, the former requires labelled data to further learn a model, which is not possible in our scenario - using the ensemble model in clinical decision makings for managing COVID-19. Therefore, a data-independent approach (like bagging) is required. For risk prediction models, their predictive capacities are underpinned by the patient characteristics of their derivation cohorts.  For example, given a new patient, models that were trained on (enough number of) similar patients likely perform better than those were not. The conventional bagging methods (majority vote or their variations) are unlikely to work very well as they are not capable of capturing such a similarity and its associations with model competence.

https://mc.manuscriptcentral.com/jamia

We propose a novel bagging mechanism using a competence assessment framework for assisting model selections in the aggregation step. The framework is designed to quantify the competence of each model for a given patient data sample. Three factors are considered. The first factor is called familiarity competence, which quantifies the above-mentioned similarity, i.e., how familiar is a model with the new patient sample to be predicted. The second factor is the general competence which can be reflected by the derivation cohort size as we know prediction models derived from large cohorts are usually superior to those from smaller ones. The final factor is to consider data completeness of a patient sample relative to a prediction model. 'Absolute' data completeness of our validation cohorts is observed to be relatively good, meaning if a clinical feature is collected at a hospital most patients tend to have it. However, 'relative' completeness (i.e., given a prediction model, the percentage of its risk predictors available in the dataset) varies significantly. Model predictive powers are likely to be compromised by such relative incompleteness, which therefore needs to be considered in the framework.

We first specify the calculation of the familiarity competence. Let $P = \{p_1, \dots, p_k\}$ be the set of all numeric predictors, $dist(m, p) = (m_p, q1_p, q3_p)$ be the distribution (median, 1st quartile and 3rd quartile respectively) of $p$ in the model $m$'s derivation cohort. Given a patient data sample: $s = \{(p,\ v_p) | p \in P\},\ where\ v_p\ is\ the\ numeric\ value\ of\ predictor\ p.$, the familiarity competence of $m$on $p$ is defined as follows.

$$C_f(s, m) = \sum_{p \in P} (1 - d(v_p, dist(m, p))),\ where\ d\ is\ a\ distance\ function\ defined\ \ as$$

$$d(v, (m, q1, q_3)) = \begin{cases} 0 & if\ q_1 \le v \le q_3, \\ min\left(\frac{|v - m|}{q3 - q1}, 1\right) & otherwise \end{cases}$$

The final competence calculation is defined as the formula below. The first component divides the familiarity competence by the total number of numeric predictors of the model, incorporating the relative data completeness of $s$ to $m$. The second component is general competence based https://mc.manuscriptcentral.com/jamia on the size of a model's derivation cohort. Assuming the two components are equally important, we calculate the overall competence as a sum of the two.

$$C = \frac{C_f(s,m)}{|P_m|} + \frac{h(m)}{\max_{m \in M}(h(m))}, \text{ where } P_m \text{ is the set of all numeric predictors of } m;\ h(m) \text{ is the derivation cohort size and } M \text{ is the set of all models.}$$

## Prediction Fusion in Ensemble Model

Different methods have been proposed in multiple classifier systems [26] to combine individual classifiers for achieving more accurate classifications. Depending on whether further training is used or not, the combination methods can be categorised as trainable combiners vs non-trainable combiners. The former (such as AdaBoost [27]) requires labelled data in the application domain (i.e., where the ensemble model is going to be used). The latter (such as majority vote combiner) can be used in a data-independent manner, i.e. applicable in new domains without the need of further training. The motivation of this work is to use the ensemble/combined model to inform decision making in care pathways or policy making, where labelled data is not available. Therefore, non-trainable combiners were used.

A set of fusion methods were implemented. For competence-independent ones, we implemented voting (majority, one positive, and one negative) and scoring (maximum and average), which are common fusion strategies used in ensemble learning [26]. When all models are assessed against the data of a given patient, the competence values can then be used to fuse predictions (probabilities of poor-prognosis) from all models. We implemented trust-the-most-competent mode: use the prediction of the one with highest competence value; wisdom-of-the-crowd mode: use the weighted average of all predictions; highest-in-top-competent-ones: use the maxim probability in top k competent models (k=3, 5). Supplementary Figure S2 gives an illustrative example of the three fusion strategies. Wisdom-of-the-crowd performed the best in our experiments and was used in this work.

https://mc.manuscriptcentral.com/jamia

The original model design is another factor that needs to be considered in the prediction fusion. Individual models were designed for predicting different severities: mortality or different definitions of severities. We manually defined a severity score for each model (death models: 1.0; poor prognosis ones: 0.3) and combined those scores in the final fusion formula as follows. The formula considers predictions from all individual models and combines them as weighted average.

$$F(s,\ M) = \begin{cases} \dfrac{\sum_{m \in M} Prob(m,s) \times C \times S_m}{|M|} & \exists m \in M, C > 0 \\ \dfrac{\sum_{m \in M} Prob(m,s) \times S_m}{|M|} & otherwise \end{cases}$$

*where* $S_m$ *is the predefined severity score of* $m$.

## Results

The performances of prediction models were evaluated on three aspects: discrimination (C-Index), model calibration and a number of parameters defining likely clinical utility. For discrimination (Figure 3a) of individual models, we observed that Xie achieved the best result (C-index 0.899, 95%CI 0.874-0.926 ) on Wuhan01, Dong performed the best (0.881, 95%CI 0.841-0.913) on Wuhan02, and Levy was the best on KCH (0.658, 95%CI 0.629-0.685) and UHB (0.660, 95%CI 0.617-0.713). None of the seven models examined consistently performed the best across all cohorts, whereas the ensemble model consistently had the best discrimination in all cases: 0.914(95%CI 0.891-0.937), 0.890(0.856-0.921), 0.665(0.640-0.692) and 0.683(0.643-0.723) on Wuhan01, Wuhan02, KCH and UHB respectively. However, the top models (Ensemble, Xie, Levy and Dong) all performed much better on Wuhan cohorts compared to the UK ones. This difference might be explained by the different admission strategies of the two countries, indicating that chosen predictors (Figure 1a.3-8) might be less predictive at later stages of clinical presentation and disease progression.

https://mc.manuscriptcentral.com/jamia

For clinical usefulness, we focus on decision-making support for admission strategies (i.e., who to admit and to where). It is not appropriate to use a fixed threshold of probability to validate model performances as (a) individual models are derived from cohorts with diverse severities and on slightly different definitions of poor prognosis; (b) severity in the validation cohorts also varies significantly. Instead, for each validation cohort we compute an event-rate (number of poor-prognosis/deceased patients divided by total number of patients) and for models we compute a prediction rate (predicted events divided by total number of patients). We then validate the sensitivity and specificity of a model when its prediction rate is closest to 1.5 times of the event rate or a minimal ratio of 0.15, whichever is larger. Figure 3b shows the performances of all models on 4 cohorts using cohort-specific prediction rate. We observed the ensemble model consistently outperforms individual models across all cohorts on positive predictive value (PPV), sensitivity and specificity. We observed prediction rate based cut-offs led to quite different performances on the metrics of PPV, sensitivity and specificity. These were what we expected. For example, for Wuhan01, the mortality rate is 2.4%, which is close to the population level. Therefore, we would expect a good model to have high specificity (ensemble model achieved 0.88) to correctly reject less severe patients so that hospital capacity can be mostly reserved for likely-to-deteriorate patients (without admitting too many mild patients). On the contrary, when the cohort is very severe (e.g. Wuhan02), high sensitivity is preferred (ensemble model: 0.96) as we don’t want to discharge those who would likely need intensive care.

To quantify how well the ensemble model reclassifies patients, we also calculated the net reclassification improvements [28] by comparing it to the best individual model on each validation cohort. Table 3 gives the details, where the ensemble model achieved net improvements in all cases with the biggest on Wuhan02 and the smallest on KCH.

We also evaluated the model calibrations of all models on all four cohorts: Figure 3c shows the calibration slope and calibration-in-large, and Figure S3 depicts the calibration plots. For individual models, similar to C-index performances, they did not perform consistently well across https://mc.manuscriptcentral.com/jamia cohorts. For example, Xie had very good calibration on Wuhan01 while it performed poorly on UHB. Again, the ensemble model has shown robust performances on all cohorts - calibrations were good to very good generally.

## Discussion

This work has shown that single models for prediction did not consistently perform well. For example, Dong's C-index on Wuhan02 is the best in individual models but it only achieved the 4$^{th}$ highest C-index on KCH. Similar situations were observed on other top single models including Xie and Levy. The challenge of getting consistent performances in diverse cohorts resides in the fact that COVID-19 prognosis will vary depending on variables underlying demography (age and comorbidity of the populations) and severities of disease in different settings (because of different admission strategies). For models derived from single cohorts, their prediction capacities were limited by the characteristics of data samples they have seen. Therefore, they are unlikely to achieve a high performance in external cohorts when there are many patients with novel characteristics. On the other hand, ensemble learning methods have the potential to make the best use of all available models. If these models were learned from complementary cohorts, the synergised model will have the potential to achieve better performances than any single model by using most competent ones for individual patients.

Comparing results in the UK (patients being admitted with more severe disease) and Chinese cohorts (more patients being admitted with mild disease), all models consistently performed worse on UK cohorts. Considering the fact that individual models used quite diverse predictors, adopted different computational algorithms and were derived from different regions/countries, it seems the observed poorer performances are likely associated with the UK's response to the first wave of COVID-19 surge. The UK mainly admitted severe patients aiming to reserve health service capacities. Therefore, one possible explanation is that blood parameters and physiological measurements are better collected as early as possible to contribute to improved predictive utility.

https://mc.manuscriptcentral.com/jamia

One limitation of this work was that we were unable to include prediction models that were learned from European cohorts, particularly from the UK. Including more local models would probably facilitate the ensemble framework to identify those predictors that are more predictive in the European cohorts, which would in turn improve the overall performance in the UK cohorts. In our future work, we will create a web platform to allow the community to share models so that a wide range of diverse and complementary models can be synergised.

## Conclusion

In this study we selected and reimplemented seven prediction models for COVID-19 with diverse derivation cohorts and different implementation techniques. A novel ensemble learning framework was proposed to synergise them for realising personalised predictions for individual patients. Four international COVID-19 cohorts were used in validating both individual and ensemble models. Validation results showed that ensemble methods could synergise a robust and good-performing model by choosing the most competent model for individual patients.

https://mc.manuscriptcentral.com/jamia

## Data sharing

Metadata of individual prediction models, their re-implementations, ensemble learning methods and all validation scripts are available at https://github.com/Honghan/EnsemblePrediction. Details of the validation cohorts are described at https://covid.datahelps.life/.

The Wuhan01 and Wuhan02 datasets used in the study will not be available due to inability to fully anonymise in line with ethical requirements. Applications for research access should be sent to TS and details will be made available via https://covid.datahelps.life/prediction/.

A subset of the KCH dataset limited to anonymisable information (e.g. only SNOMED codes and aggregated demographics) is available on request to researchers with suitable training in information governance and human confidentiality protocols subject to approval by the King's College Hospital Information Governance committee; applications for research access should be sent to kch-tr.cogstackrequests@nhs.net. This dataset cannot be released publicly due to the risk of re-identification of such granular individual level data, as determined by the King's College Hospital Caldicott Guardian.

A subset of the UHB dataset limited to aggregate anonymised information is available on request to researchers with suitable training in information governance and human confidentiality protocols, subject to approval and data sharing agreements by the UHB hospitals NHS foundation trust.

## Ethics approval and consent to participate

The KCH component of the project operated under London South East Research Ethics Committee (reference 18/LO/2048) approval granted to the King’s Electronic Records Research Interface (KERRI); specific work on COVID-19 research was reviewed with expert patient input on a virtual committee with Caldicott Guardian oversight. The UHB validation was performed as part of a service evaluation agreed with approval from trust research leads and the Caldicott Guardian. The Wuhan validations were approved by the Research Ethics Committee of Shanghai Dongfang Hospital and Taikang Tongji Hospital.

## Competing interest statement

We declare no competing interests

## Acknowledgements

HW and HZ are supported by Medical Research Council and Health Data Research UK Grant (MR/S004149/1), Industrial Strategy Challenge Grant (MC_PC_18029) and Wellcome Institutional Translation Partnership Award (PIII054). AK is supported by Medical Research Council and Health Data Research UK Grant (MR/S003991/1). XW is supported by the National Natural Science Foundation of China (grant number 81700006). DMB is funded by a UKRI Innovation Fellowship (Health Data Research UK MR/S00310X/1).TV/FGS/TW are funded by NIHR covid/non-covid research grants and Queen Elizabeth Hospital Charities. KD is supported by the LifeArc STOPCOVID award. VRC and GVG acknowledge support from the NIHR Birmingham ECMC, NIHR Birmingham SRMRC, Nanocommons H2020-EU (731032) and the NIHR Birmingham Biomedical Research Centre and the MRC Health Data Research UK

https://mc.manuscriptcentral.com/jamia

(HDRUK/CFC/01). RJBD is supported by: (1) NIHR Biomedical Research Centre at South London and Maudsley NHS Foundation Trust and King's College London, London, U.K. (2) Health Data Research UK (3) The BigData@Heart Consortium, funded by the Innovative Medicines Initiative-2 Joint Undertaking under grant agreement No. 116074. (4) The National Institute for Health Research University College London Hospitals Biomedical Research Centre. (5) The UK Research and Innovation London Medical Imaging & Artificial Intelligence Centre for Value Based Healthcare (6) the National Institute for Health Research (NIHR) Applied Research Collaboration South London (NIHR ARC South London) at King's College Hospital NHS Foundation Trust. This work uses data provided by patients and collected by the NHS as part of their care and support. We thank Prof Ye Yan (from Huazhong University of Science and Technology, Wuhan, China) for his support of providing access to the Wuhan02 cohort.

## Author contributions

HW, HZ, ZI, RD and BG conceived the study design and developed the study objectives. ZI, HZ, TS contributed to the statistical analyses. KD provided overall clinical input to the study. HW performed the model reimplementation, ensemble learning and software development. For KCH data, DB, JT were responsible for the data extraction and preparation; JT, KO, RZ provided clinical input; JT performed data validation. For UHB data, AK, VRG, TV were responsible for data extraction and preparation; FGS, TW, TV and GVG provided clinical input and validated the results on UHB data. For the Wuhan01 cohort, XW, XZ, XW and JS extracted the data from the EHR system. HW and HZ preprocessed the raw data and conducted the prediction model validations; BG, HW, HZ, TS and JS interpreted the data and results. For Wuhan02, Prof Ye Yan, KL were responsible for data extraction and preparation; KL, HW conducted the prediction model validations; YY interpreted the data and results. All authors contributed to the interpretation of the data, critical revision of the manuscript, and approved the final version of the manuscript.

https://mc.manuscriptcentral.com/jamia

## Tables

| | Wuhan01 cohort (n=2869) | | Wuhan02 cohort (n=357) | | KCH cohort (n=1475) | | UHB cohort (n=693) | |
|---|---|---|---|---|---|---|---|---|
| | **Not poor prog. (n=2738)** | **Poor prog. (n=131)** | **Did not die (n=194)** | **Died (n=163)** | **Not poor prog. (n=949)** | **Poor prog. (n=526)** | **Not poor prog. (n=477)** | **Poor prog. (n=216)** |
| Age | 60 (49-68) | 70 (63-78) | 51 (37-62) | 69 (62-77) | 69 (54-81) | 75 (60-86) | 72 (57-82) | 70 (56-80) |
| Sex (male) | 1389 (50.7%) | 84 (64.1%) | 91 (46.9%) | 118 (72.4%) | 514 (54.2%) | 330 (62.7%) | 254 (53.2%) | 144 (66.7%) |
| **Clinical features - median (IQR)** | | | | | | | | |
| Red cell distribution width | 12.9 (12.3-13.5) | 13.0 (12.5-14.0) | 12.0 (11.8-12.7) | 12.9 (12.3-13.9) | - | - | 13.7 (12.7-15.4) | 13.9 (13.2-15.1) |
| Albumin | 38.3 (35.5-40.7) | 31.6 (28.7-35.0) | 37.5 (34.2-40.2) | 30.1 (27.6-33.0) | 38.0 (35.0-41.0) | 36.0 (33.0-39.0) | 31.0 (26.0-35.0) | 28.0 (22.0-32.0) |
| C-reactive protein | 2.1 (0.8-7.3) | 59.9 (14.2-120.0) | 19.5 (3.8-49.8) | 114.1 (61.9-178.8) | 72.5 (28.8-127.9) | 112.2 (56.8-216.5) | 83.0 (42.0-140.2) | 180.0 (102.5-267.0) |
| Serum blood urea nitrogen | 4.3 (3.6-5.4) | 6.8 (5.0-11.0) | - | - | - | - | 6.3 (4.5-10.4) | 8.1 (5.4-13.1) |
| Lymphocyte count | 1.5 (1.1-1.9) | 0.7 (0.5-1.1) | 1.1 (0.8-1.5) | 0.6 (0.4-0.8) | 1.0 (0.7-1.4) | 0.9 (0.6-1.4) | 0.9 (0.7-1.3) | 0.9 (0.6-1.2) |
| Direct bilirubin | 3.3 (2.5-4.4) | 5.4 (3.5-7.2) | 3.5 (2.5-4.7) | 6.2 (4.4-9.2) | - | - | 10.0 (7.0-14.0) | 11.0 (8.0-20.0) |
| Lactate dehydrogenase | 174.6 (150.3-210.2) | 332.2 (244.9-461.0) | 250.0 (202.2-310.5) | 567.0 (427.5-762.0) | - | - | 316.5 (245.8-411.0) | 436.0 (340.0-623.0) |
| Serum sodium | 141.6 (140.0-143.2) | 139.8 (137.4-143.4) | 139.2 (136.5-141.2) | 138.9 (135.8-143.6) | - | - | 137.0 (134.0-140.0) | 138.0 (135.0-143.0) |
| Neutrophil count | 3.5 (2.7-4.5) | 6.7 (4.8-9.9) | - | - | 5.1 (3.7-7.4) | 6.6 (4.5-9.4) | 4.7 (3.4-6.7) | 6.7 (4.8-9.4) |
| Oxygen saturation | 97.8 (97.0-98.2) | 96.6 (94.5-97.7) | - | - | 19 (18-20) | 23 (20-28) | 94.0 (93.0-96.0) | 92.0 (88.0-94.0) |

Table 1. The baselines of poor prognosis/death subgroups vs not poor prognosis/survival subgroups of 4 cohorts. Data are median (IQR) or number (%). Poor prognosis is defined as either ICU stay or death. Wuan02 does not have ICU stay data, therefore its analysis only compared death/survival instead.

https://mc.manuscriptcentral.com/jamia

| | Shi | Xie | Dong | Levy | Yan | Gong | Lu |
|---|---|---|---|---|---|---|---|
| outcome | Poor prognosis | Death | Poor prognosis | Death | Death | Poor prognosis | Death |
| model type | scoring | Logistic regression | nomogram | NOCOS | Decision tree | nomogram | scoring |
| Region | Zhejiang | Wuhan | Anhui, Beijing | New York | Wuhan | Wuhan, Guangzhou | Wuhan |
| Derivation cohort size | 487 | 299 | 208 | 11,095 | 375 | 189 | 577 |
| Age - median [IQR] | 46 [27-65] | 65 [54-73] | 44 [28-60] | 65 [54-77] | 59 [42-75] | 49 [35-63] | 55 [39-66] |
| Follow-up period (in 2020) | Unknown to Feb 17 | Jan 01 to Feb01 | Jan 20 to Mar 18 | Mar 01 to May 05 | Jan 10 to Feb18 | Jan 20 to Mar 02 | Jan 21 to Feb 05 |
| Mortality rate | - | 51.84% | - | 23.40% | 41.33% | - | 6.76% |
| Poor prognosis rate | 10.06% | - | 19.23% | - | - | 14.81% | 17.33% |

Table 2. Seven prognosis prediction models. For outcomes, poor prognosis is defined as severities including length of stay, ICU stay, or categories of treatments. For model type, scoring - models that calculate a sum from scores predefined to individual predictor values; logistic regression and decision tree - models where these computational models are used; nomogram - models represented as a 2-dimensional graphical calculating diagram. NOCOS - a customised model.

| | Wuhan01 (Ensemble vs Xie) | | Wuhan02 (Ensemble vs Dong) | | KCH (Ensemble vs Levy) | | UHB (Ensemble vs Levy) | |
|---|---|---|---|---|---|---|---|---|
| | Event | No Event | Event | No Event | Event | No Event | Event | No Event |
| Higher | 13 | 132 | 26 | 10 | 51 | 77 | 15 | 42 |
| Lower | 7 | 124 | 16 | 17 | 48 | 74 | 11 | 37 |
| Total | 432 | 2,438 | 127 | 230 | 642 | 833 | 325 | 368 |

https://mc.manuscriptcentral.com/jamia

| | | | | |
|---|---|---|---|---|
| Net Reclassification Improvements | 1.72% | 4.83% | 0.83% | 2.59% |

Table 3. Net reclassification improvements of Ensemble model compared to the best individual model on each validation cohort.

## Figure captions

**Figure 1** Validation cohorts, prognosis models and ensemble learning. **a,** four validation cohorts. a.1 - cohort size and mortalities; a.2 - follow-ups aligned with wave 1 periods of China and the UK, red colours indicating high new daily cases; a.3 - age distributions; a.4-7 - distributions of bloods and vitals. **b,** timeline of follow-up periods of derivation cohorts of all individual prediction models. **c,** Illustrative diagram of ensemble learning by combining three linear models for binary classification.

**Figure 2** Architecture of the proposed ensemble learning framework. At the centre is the ensemble method taking seven individual models as input (top left) and synergising them based on their competence on target cohorts. Four international COVID-19 cohorts (top right) were included in this study for evaluation of ensemble learning (bottom).

**Figure 3** Validation results of discrimination, clinical usefulness and calibration. **a,** discrimination performances: median (95%CI). **b,** positive predictive value (PPV), sensitivity and specificity of all models validated on cohort-specific prediction rate. Models that could not achieve expected prediction rates were excluded. **c,** calibration results on four validation cohorts: median (95%CI) where empty cells are for those models which were not validated because they were derived from the same hospital data.

https://mc.manuscriptcentral.com/jamia

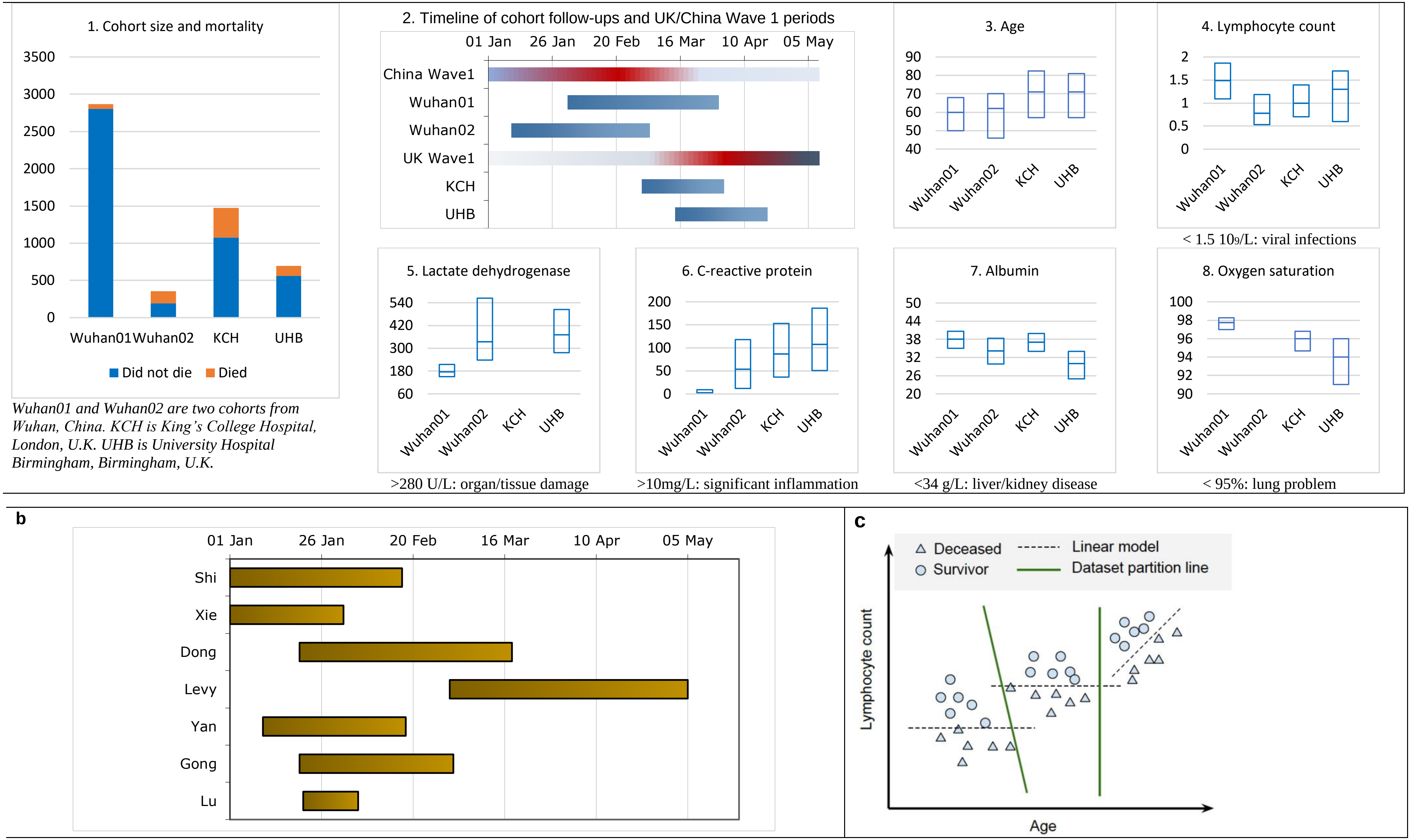

1. Cohort size and mortality
3500
3000
2500
2000
1500
1000
500
0
Wuhan01
Wuhan02
KCH
UHB
Did not die
Died
Wuhan01 and Wuhan02 are two cohorts from Wuhan, China. KCH is King's College Hospital, London, U.K. UHB is University Hospital Birmingham, Birmingham, U.K.
2. Timeline of cohort follow-ups and UK/China Wave 1 periods
01 Jan
26 Jan
20 Feb
16 Mar
10 Apr
05 May
China Wave1
Wuhan01
Wuhan02
UK Wave1
KCH
UHB
3. Age
90
80
70
60
50
40
4. Lymphocyte count
2
1.5
1
0.5
0
< 1.5 $10^9$/L: viral infections
5. Lactate dehydrogenase
540
420
300
180
60
>280 U/L: organ/tissue damage
6. C-reactive protein
200
150
100
50
0
>10mg/L: significant inflammation
7. Albumin
50
44
38
32
26
20
<34 g/L: liver/kidney disease
8. Oxygen saturation
100
98
96
94
92
90
< 95%: lung problem
b
01 Jan
26 Jan
20 Feb
16 Mar
10 Apr
05 May
Shi
Xie
Dong
Levy
Yan
Gong
Lu
c
Deceased
Survivor
Linear model
Dataset partition line
Lymphocyte count

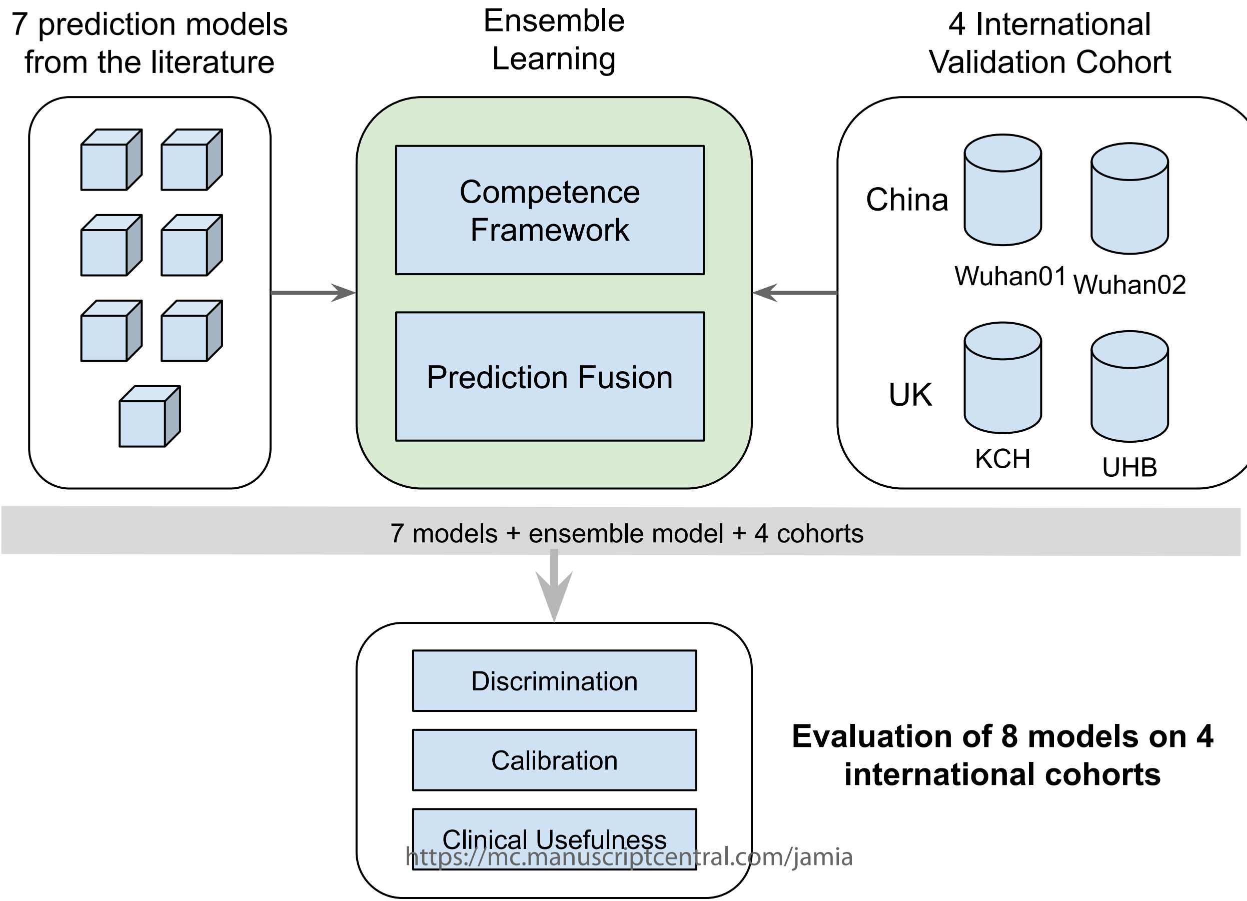

Age https://mc.manuscriptcentral.com/jamia

**a**

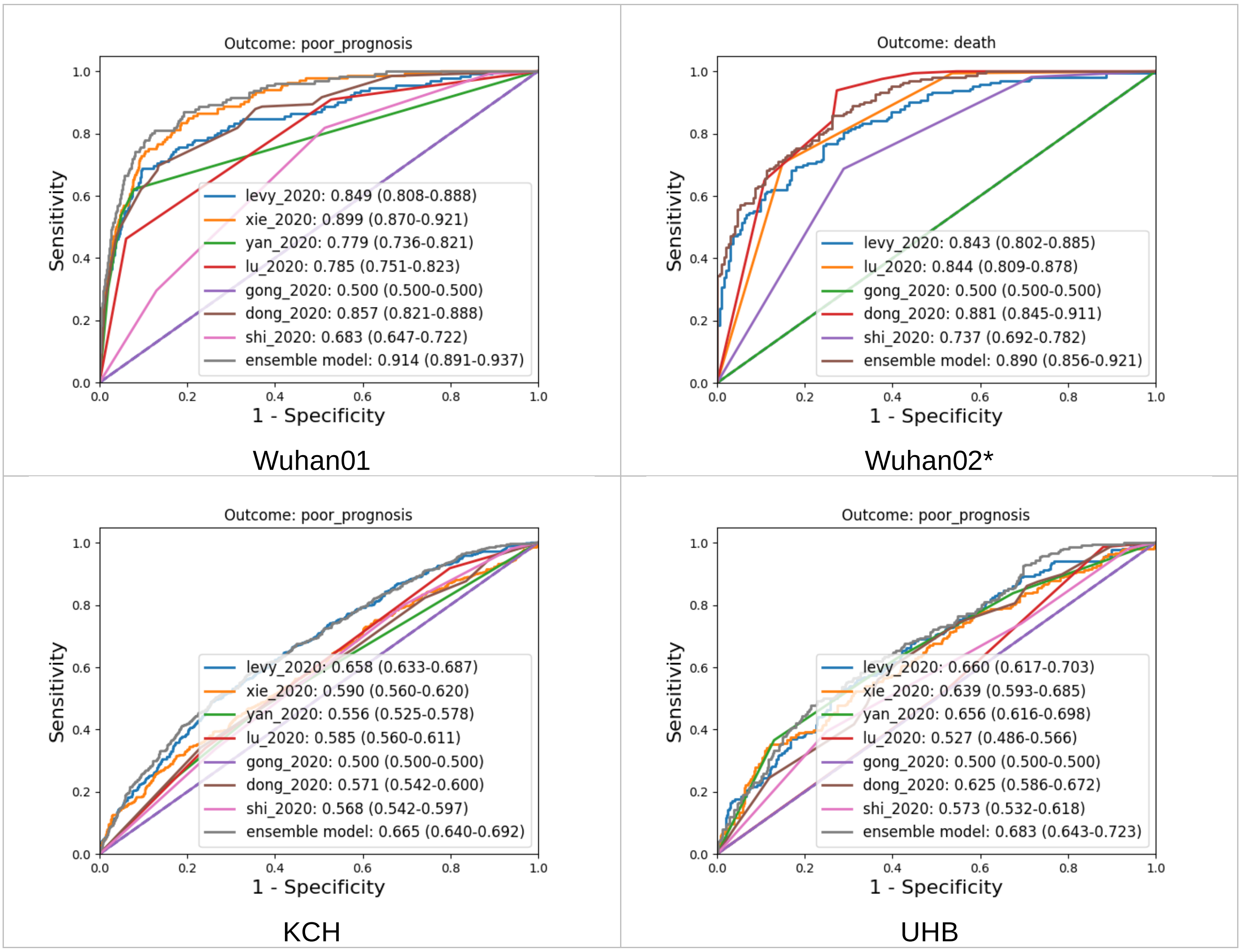

* Yan/Shi were not evaluated on Wuhan02 as they were derived from the same hospital data

**b**

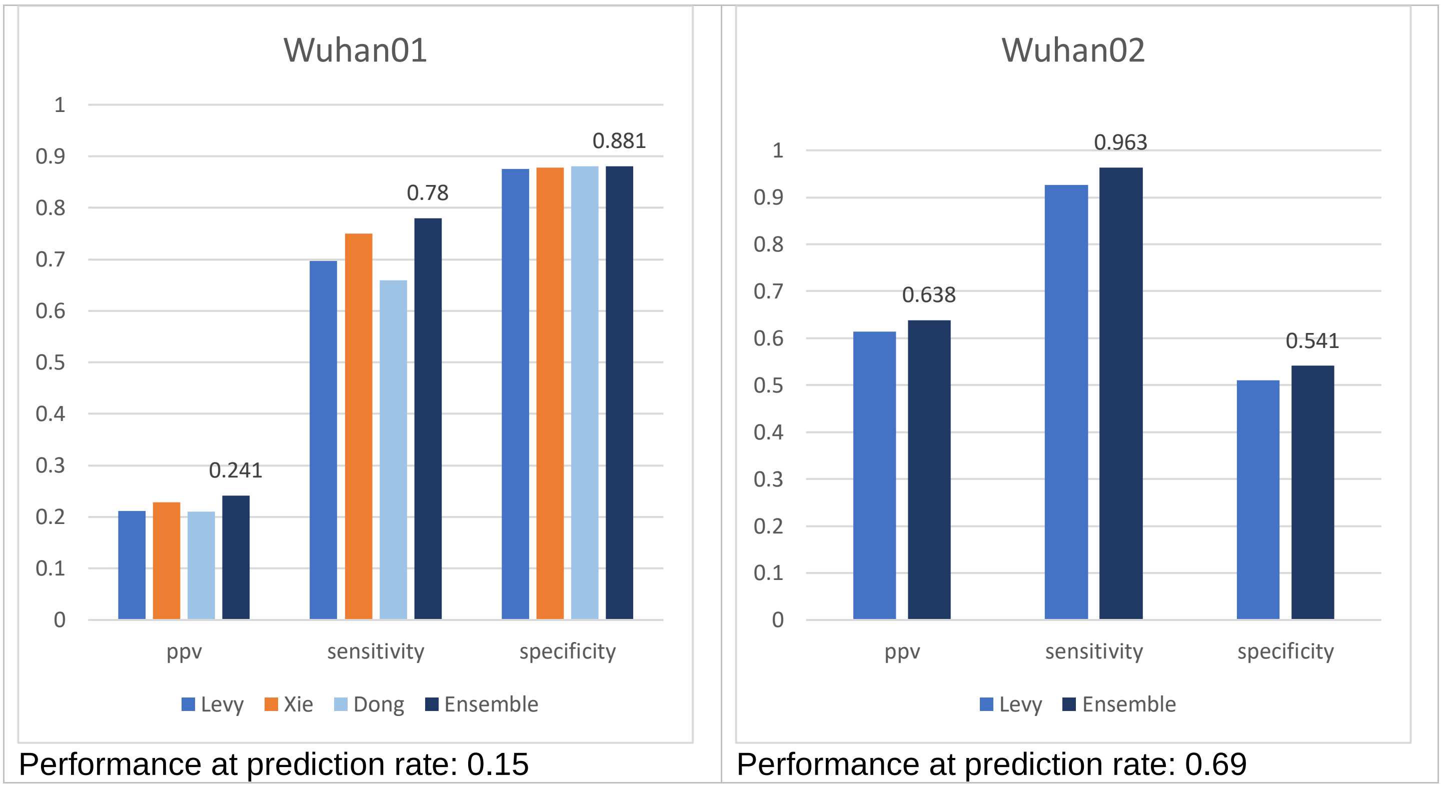

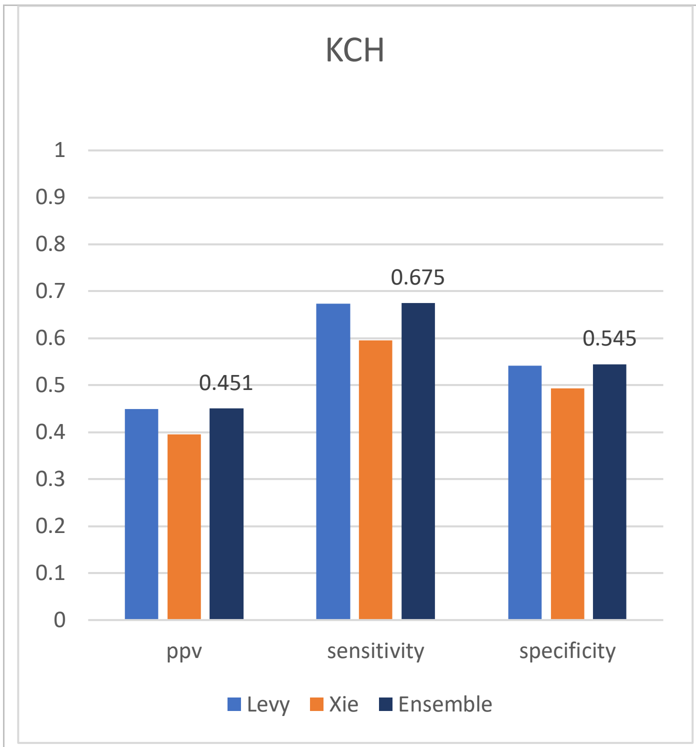

Performance at prediction rate: 0.54

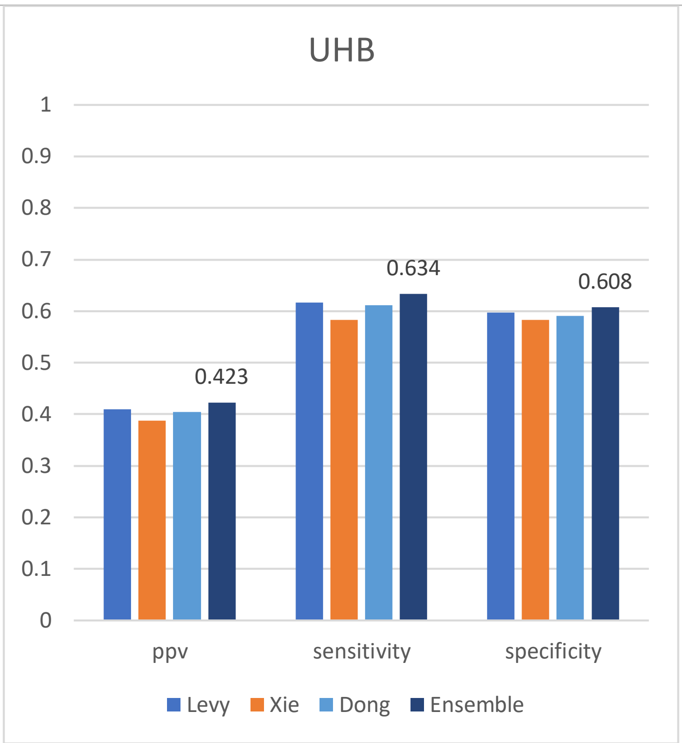

Performance at prediction rate: 0.47

**c**

**Calibration results on four validation cohorts**

| | Levy | Xie | Yan | Lu | Gong | Dong | Shi | Ensemble model |
|---|---|---|---|---|---|---|---|---|
| Wuhan01 (Poor prognosis) | | | | | | | | |
| **slope** | 0.952 (0.952-0.952) | 1.124 (1.124-1.000) | 0.482 (0.482-0.482) | 0.807 (0.807-0.807) | 0.000 (0.000-0.000) | 0.405 (0.405-0.405) | 0.235 (0.235-0.235) | 1.462 (1.462-1.000) |
| **calibration-in-large** | 0.127 (0.127-0.127) | -0.050 (0.000--0.050) | -0.026 (0.000--0.026) | 0.002 (0.002-0.002) | 0.046 (0.046-0.046) | -0.047 (0.000--0.047) | -0.015 (0.000--0.015) | -0.022 (0.000--0.022) |
| Wuhan02 (Death) | | | | | | | | |
| **slope** | 0.843 (0.843-0.843) | | | 1.939 (1.939-1.000) | 0.000 (0.000-0.000) | 1.232 (1.232-1.000) | 1.759 (1.759-1.000) | 1.214 (1.214-1.000) |
| **calibration-in-large** | 0.572 (0.572-0.572) | | | 0.159 (0.159-0.159) | 0.457 (0.457-0.457) | 0.047 (0.047-0.047) | 0.004 (0.004-0.004) | 0.477 (0.477-0.477) |
| KCH (Poor prognosis) | | | | | | | | |
| **slope** | 0.958 (0.958-0.958) | 0.987 (0.987-0.987) | 0.232 (0.232-0.232) | 0.401 (0.401-0.401) | 0.000 (0.000-0.000) | 0.276 (0.276-0.276) | 0.625 (0.625-0.625) | 1.061 (1.061-1.000) |
| **calibration-in-large** | 0.221 (0.221-0.221) | 0.161 (0.161-0.161) | 0.304 (0.304-0.304) | 0.306 (0.306-0.306) | 0.357 (0.357-0.357) | 0.276 (0.276-0.276) | 0.144 (0.144-0.144) | 0.196 (0.196-0.196) |
| UHB (Poor prognosis) | | | | | | | | |
| **slope** | 0.587 (0.587-0.587) | 0.668 (0.668-0.668) | 0.415 (0.415-0.415) | 0.010 (0.010-0.010) | 0.000 (0.000-0.000) | 0.266 (0.266-0.266) | 0.497 (0.497-0.497) | 0.933 (0.933-0.933) |
| **calibration-in-large** | 0.197 (0.197-0.197) | 0.124 (0.124-0.124) | 0.123 (0.123-0.123) | 0.310 (0.310-0.310) | 0.312 (0.312-0.312) | 0.255 (0.255-0.255) | 0.148 (0.148-0.148) | 0.067 (0.067-0.067) |
