# Ensemble learning for poor prognosis predictions: A case study on SARS-CoV-2

**DOI:** 10.1093/jamia/ocaa295

**Published:** 2020-11-13

**Authors:** Honghan Wu, Huayu Zhang, Andreas Karwath, Zina Ibrahim, Ting Shi, Xin Zhang, Kun Wang, Jiaxing Sun, Kevin Dhaliwal, Daniel Bean, Victor Roth Cardoso, Kezhi Li, James T Teo, Amitava Banerjee, Fang Gao-Smith, Tony Whitehouse, Tonny Veenith, Georgios V Gkoutos, Xiaodong Wu, Richard Dobson, Bruce Guthrie

**Affiliations:** 1 Institute of Health Informatics, University College London, London, United Kingdom; 2 Health Data Research UK, University College London, London, United Kingdom; 3 Centre for Medical Informatics, Usher Institute, University of Edinburgh, Edinburgh, United Kingdom; 4 Institute of Cancer and Genomic Sciences, University of Birmingham, Birmingham, United Kingdom; 5 Health Data Research UK, University of Birmingham, Birmingham, United Kingdom; 6 Department of Biostatistics and Health Informatics, Institute of Psychiatry, Psychology and Neuroscience, King’s College London, London, United Kingdom; 7 Centre for Global Health, Usher Institute, University of Edinburgh, Edinburgh, United Kingdom; 8 Department of Pulmonary and Critical Care Medicine, People’s Liberation Army Joint Logistic Support Force 920th Hospital, Kunming, China; 9 Department of Pulmonary and Critical Care Medicine, Shanghai East Hospital, Tongji University, Shanghai, China; 10 Centre for Inflammation Research, Queens Medical Research Institute, University of Edinburgh, Edinburgh, United Kingdom; 11 Department of Stroke and Neurology, King’s College Hospital NHS Foundation Trust, London, United Kingdom; 12 Department of Intensive Care Medicine, Queen Elizabeth Hospital Birmingham, Birmingham, United Kingdom; 13 Birmingham Acute Care Research, University of Birmingham, Birmingham, United Kingdom; 14 Institute of Translational Medicine, University Hospitals Birmingham NHS Foundation Trust, Birmingham, United Kingdom; 15 Department of Pulmonary and Critical Care Medicine, Taikang Tongji Hospital, Wuhan, China; 16 Centre for Population Health Sciences, Usher Institute, University of Edinburgh, Edinburgh, United Kingdom

**Keywords:** ensemble learning, model synergy, risk prediction, COVID-19, decision support

## Abstract

**Objective:**

Risk prediction models are widely used to inform evidence-based clinical decision making. However, few models developed from single cohorts can perform consistently well at population level where diverse prognoses exist (such as the SARS-CoV-2 [severe acute respiratory syndrome coronavirus 2] pandemic). This study aims at tackling this challenge by synergizing prediction models from the literature using ensemble learning.

**Materials and Methods:**

In this study, we selected and reimplemented 7 prediction models for COVID-19 (coronavirus disease 2019) that were derived from diverse cohorts and used different implementation techniques. A novel ensemble learning framework was proposed to synergize them for realizing personalized predictions for individual patients. Four diverse international cohorts (2 from the United Kingdom and 2 from China; N = 5394) were used to validate all 8 models on discrimination, calibration, and clinical usefulness.

**Results:**

Results showed that individual prediction models could perform well on some cohorts while poorly on others. Conversely, the ensemble model achieved the best performances consistently on all metrics quantifying discrimination, calibration, and clinical usefulness. Performance disparities were observed in cohorts from the 2 countries: all models achieved better performances on the China cohorts.

**Discussion:**

When individual models were learned from complementary cohorts, the synergized model had the potential to achieve better performances than any individual model. Results indicate that blood parameters and physiological measurements might have better predictive powers when collected early, which remains to be confirmed by further studies.

**Conclusions:**

Combining a diverse set of individual prediction models, the ensemble method can synergize a robust and well-performing model by choosing the most competent ones for individual patients.

## INTRODUCTION

Risk prediction models are widely used in clinical practice to inform decision making.^1–3^ Good models cannot only improve health service efficiencies, but also predict deterioration^4^ in a proactive manner,^5^ with a great potential to improve outcomes and save lives. Such evidence-based decision making supports are particularly important in an epidemic or pandemic outbreak, not only for informing the treatments or managements of those infected, but also for optimizing healthcare services to minimize indirect effects to most vulnerable service users. For example, the recent severe acute respiratory syndrome coronavirus 2 (SARS-CoV-2) has caused substantial excess mortality,^6^^,^^7^ at least partly due to an indirect effect on healthcare systems, leading to a loss of capacity to provide elective and emergency care within the “golden window” of opportunity.^7–9^ To mitigate excess mortality, more targeted inpatient care in future waves could be informed by (1) better risk prediction and (2) insights from international coronavirus disease 2019 (COVID-19) (we use the terms *SARS-CoV-2* and *COVID-19* interchangeably) datasets and experience to validate models and learn from different countries’ responses.

**Figure 1.**
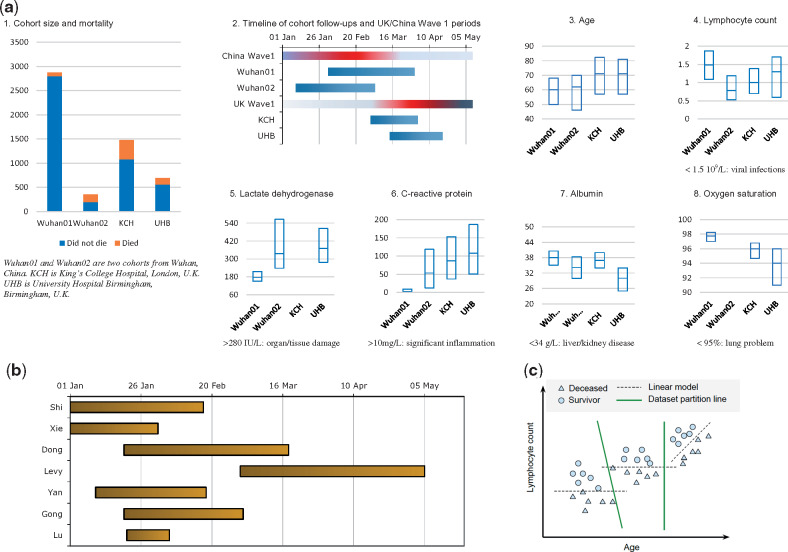
Validation cohorts, prognosis models, and ensemble learning. **(A)** The 4 validation cohorts. A.1 shows cohort size and mortalities; A.2 shows follow-ups aligned with wave 1 periods of China and the United Kingdom (red indicates high new daily cases); A.3 shows age distributions; A.4-A.7 show distributions of bloods and vitals. **(B)** Timeline of follow-up periods of derivation cohorts of all individual prediction models. **(C)** Illustrative diagram of ensemble learning by combining 3 linear models for binary classification. KCH: King’s College Hospital; UHB: University Hospitals Birmingham.

There have been numerous prediction models developed for COVID-19,^10–14^ but most were derived in small datasets, had low methodological quality, and are unvalidated.^13^ In addition, models learned from single cohorts (even from several centers) might not have the predictive power to achieve good performance in situations in which a disease spreads to the whole population, leading to greatly diverse prognoses. In this study, we reproduced various prediction models with reasonable quality and synergized them using ensemble learning^15^ to assess their collective ability to accurately discriminate mild and severe patients in a diverse set of 4 patient cohorts from the United Kingdom and China with varying patterns of disease severity (Figure 1A). In particular, China and the United Kingdom had very different approaches to hospital admission for COVID-19. In Wuhan, admission was routine with patients triaged to low intensity (Fangcang hospitals)^16^ or higher dependency (designated hospitals) settings, whereas in the United Kingdom, admission of patients with more severe disease or at perceived higher risk of severe disease was prioritized. These differences enabled us to assess model performance in different settings. For outcomes specifically, we primarily focused on poor prognosis defined by either death or intensive care unit stay.

## MATERIALS AND METHODS

**Figure 2.**
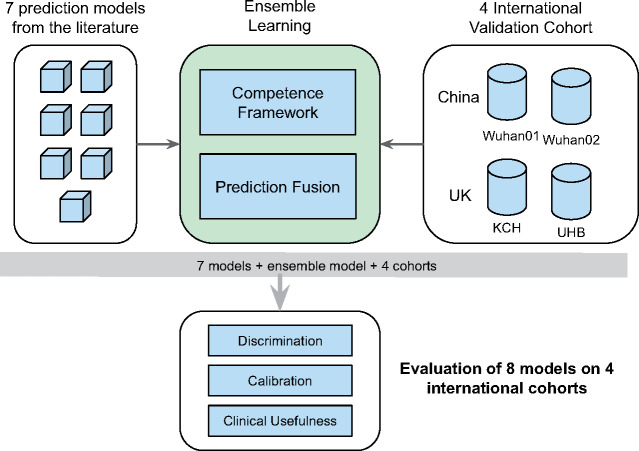
Architecture of the proposed ensemble learning framework. At the center is the ensemble method taking 7 individual models as input (top left) and synergizing them based on their competence on target cohorts. Four international COVID-19 cohorts (top right) were included in this study for evaluation of ensemble learning (bottom). KCH: King’s College Hospital; UHB: University Hospitals Birmingham.

Figure 2 depicts the architecture of this work—synergizing individual models from the literature for preventing excess mortality. For prediction models (Figure 1B), 7 models—Dong,^10^ Shi,^17^ Gong,^18^ Lu,^19^ Yan,^20^ Xie,^21^ and Levy^22^—were chosen with different model types using diverse sets of predictors. Derivation cohorts were diverse, originating from 6 regions in 2 countries, with median ages ranging from 44 to 65 years, and with mortality varying between 7% and 52%. Such diversity provides leverage for synergizing insights from these derivation cohorts to obtain a collective and hopefully improved predictive power.

To synergize models derived from multinational datasets, we used ensemble learning,^15^^,^^23^ a machine learning methodology that is particularly effective when single models perform well at certain subsets of the whole data samples but none of them can achieve good overall performances. The rationale is to partition the data samples into groups and choose the most suitable model(s) for particular groups (eg, to give more weights to models derived from older populations with more severe cases for a 78-year-old patient with lymphocyte count of 0.7) so that the optimal overall prediction result can be achieved. Figure 1C shows a synthetic and schematic illustration of such a situation. In (conventional) ensemble learning scenarios, weak predictors are usually trained on subsets of the same dataset. The key difference of this work is that the weak predictors were not trained locally on one particular dataset, but rather were selected from the literature (ie, learned from external datasets, which the ensemble model does not have access to) and reimplemented for aggregation.

The aggregation approaches used in this study do not belong to the stacking method (also called stacked generalization),^24^ that is to learn a new model using inputs from individual classifiers. Instead, they are inspired by bagging predictors^25^—aggregating results in a data-independence manner.

### Validation and analytics cohorts

The first Wuhan cohort (Wuhan01) consisted of 2869 adults with COVID-19 confirmed by reverse transcriptase polymerase chain reaction admitted to 1 of 2 hospitals in Wuhan, China (Wuhan Sixth Hospital and Taikang Tongji Hospital), admitted between February 1 and 23, 2020, and who died or were discharged on or before March 29, 2020. The second Wuhan cohort (Wuhan02) consisted of 357 adults with COVID-19 from Tongji Hospital, data of which was collected between January 1 and March 4, 2020. The first UK cohort (King's College Hospital [KCH]) consisted of 1475 adults (≥18 years of age) hospitalized with COVID-19 in King’s College Hospital NHS Foundation Trust (London, United Kingdom) between March 1 and April 2, 2020, who were followed up until April 8, 2020. The second UK cohort (University Hospitals Birmingham [UHB]) consisted of 693 adults (≥18 years of age) hospitalized with COVID-19 at the Queen Elizabeth Hospital (part of the University Hospital Trust, Birmingham, United Kingdom) between March 14 and April 13, 2020, who were followed up to April 19, 2020. Mortality rates of Wuhan01, Wuhan02, KCH, and UHB are 2.4%, 45.7%, 26.9%, and 19.0%, respectively. The large difference in mortality between 2 Wuhan cohorts was possibly because (1) Wuhan02 admitted more severe cases under Wuhan city-wide coordination^20^ and (2) the 2 were followed up in different periods related to the surge (Figure 1A.2). Table 1 gives the baseline for comparing poor prognosis or died and not poor prognosis and did not die subgroups of all 4 cohorts. All cohorts were retrospective and extracted from electronic health records for this study. Demographics and baselines of all 4 validation cohorts are described in detail in Supplementary Tables S2-S5.

**Table 1.** The baselines of poor prognosis and death subgroups vs not poor prognosis and survival subgroups of 4 cohorts.

	Wuhan01 Cohort (n = 2869)		Wuhan02 Cohort (n = 357)		KCH Cohort (n = 1475)		UHB Cohort (n = 693)	
	Not Poor Prognosis (n = 2738)	Poor Prognosis (n = 131)	Did Not Die (n = 194)	Died (n = 163)	Not Poor Prognosis (n = 949)	Poor Prognosis (n = 526)	Not Poor Prognosis (n = 477)	Poor Prognosis (n = 216)
Age, y	60 (49-68)	70 (63-78)	51 (37-62)	69 (62-77)	69 (54-81)	75 (60-86)	72 (57-82)	70 (56-80)
Male	1389 (50.7)	84 (64.1)	91 (46.9)	118 (72.4)	514 (54.2)	330 (62.7)	254 (53.2)	144 (66.7)
**Clinical features**								
Red cell distribution width (percentage)	12.9 (12.3-13.5)	13.0 (12.5-14.0)	12.0 (11.8-12.7)	12.9 (12.3-13.9)	–	–	13.7 (12.7-15.4)	13.9 (13.2-15.1)
Albumin (g/L)	38.3 (35.5-40.7)	31.6 (28.7-35.0)	37.5 (34.2-40.2)	30.1 (27.6-33.0)	38.0 (35.0-41.0)	36.0 (33.0-39.0)	31.0 (26.0-35.0)	28.0 (22.0-32.0)
C-reactive protein (mg/L)	2.1 (0.8-7.3)	59.9 (14.2-120.0)	19.5 (3.8-49.8)	114.1 (61.9-178.8)	72.5 (28.8-127.9)	112.2 (56.8-216.5)	83.0 (42.0-140.2)	180.0 (102.5-267.0)
Serum blood urea nitrogen (mmol/L)	4.3 (3.6-5.4)	6.8 (5.0-11.0)	–	–	–	–	6.3 (4.5-10.4)	8.1 (5.4-13.1)
Lymphocyte count (10^9^/L)	1.5 (1.1-1.9)	0.7 (0.5-1.1)	1.1 (0.8-1.5)	0.6 (0.4-0.8)	1.0 (0.7-1.4)	0.9 (0.6-1.4)	0.9 (0.7-1.3)	0.9 (0.6-1.2)
Direct bilirubin (umol/L)	3.3 (2.5-4.4)	5.4 (3.5-7.2)	3.5 (2.5-4.7)	6.2 (4.4-9.2)	–	–	10.0 (7.0-14.0)	11.0 (8.0-20.0)
Lactate dehydrogenase (IU/L)	174.6 (150.3-210.2)	332.2 (244.9-461.0)	250.0 (202.2-310.5)	567.0 (427.5-762.0)	–	–	316.5 (245.8-411.0)	436.0 (340.0-623.0)
Serum sodium (mmol/L)	141.6 (140.0-143.2)	139.8 (137.4-143.4)	139.2 (136.5-141.2)	138.9 (135.8-143.6)	–	–	137.0 (134.0-140.0)	138.0 (135.0-143.0)
Neutrophil count (10^9^/L)	3.5 (2.7-4.5)	6.7 (4.8-9.9)	–	–	5.1 (3.7-7.4)	6.6 (4.5-9.4)	4.7 (3.4-6.7)	6.7 (4.8-9.4)
Oxygen saturation (percentage)	97.8 (97.0-98.2)	96.6 (94.5-97.7)	–	–	19 (18-20)	23 (20-28)	94.0 (93.0-96.0)	92.0 (88.0-94.0)

Values are median (interquartile range) or n (%). Poor prognosis is defined as either intensive care unit stay or death. Wuan02 does not have intensive care unit stay data; therefore, its analysis only compared death/survival instead.

KCH: King’s College Hospital; UHB: University Hospitals Birmingham.

### Prediction model selection and reimplementation

In May 2020, we conducted a literature search for COVID-19 poor prognosis models. The search and selection process are described with details in Supplementary Figure S1. Briefly, for prediction models (Figure 1B), we selected COVID-19 prognosis (either death or severity) models that were (1) reproducible (implementable models with all parameters reported); (2) using predictors that are readily available at community triage at large scale (ie, demographics, underlying conditions, blood tests, and vital signs); and (3) with sufficient information describing the derivation cohort including cohort size, interquartile range of age, country/region, follow-up period, and mortality and poor prognosis ratios. Table 2 describes information of the 7 models including the outcomes, computational methods, information of derivation cohorts (eg, size, region or country, mortality rate, follow-up period).

**Table 2.** Seven prognosis prediction models.

	Shi	Xie	Dong	Levy	Yan	Gong	Lu
Outcome	Poor prognosis	Death	Poor prognosis	Death	Death	Poor prognosis	Death
Model type	Scoring	Logistic regression	Nomogram	NOCOS^a^	Decision tree	Nomogram	Scoring
Region	Zhejiang	Wuhan	Anhui, Beijing	New York	Wuhan	Wuhan, Guangzhou	Wuhan
Derivation cohort size	487	299	208	11,095	375	189	577
Age, y	46 (27-65)	65 (54-73)	44 (28-60)	65 (54-77)	59 (42-75)	49 (35-63)	55 (39-66)
Follow-up period (in 2020)	Unknown to February 17	January 1 to Feb01	January 20 to March 18	March 01 to May 05	January 10 to February 18	January 20 to March 02	January 21 to February 05
Mortality rate	–	51.84%	–	23.40%	41.33%	–	6.76%
Poor prognosis rate	10.06%	–	19.23%	–	–	14.81%	17.33%

Values are median (interquartile range) or n (%). For outcomes, poor prognosis is defined as severities including length of stay, intensive care unit stay, or categories of treatments. For model type, scoring refers to models that calculate a sum from scores predefined to individual predictor values; logistic regression and decision tree refers to models in which these computational models are used; nomogram refers to models represented as a 2-dimensional graphical calculating diagram. ^a^Customized model.

We reimplemented these 7 prediction models by extracting all parameters from their published or preprint manuscripts or public-facing websites. Five different models are implemented including decision tree, logistic regression, nomogram, scoring, and NOCOS (a customized transparent model). We also extracted derivation cohort size, follow-up periods, and distributions of numeric predictors (bloods and vitals). Supplementary Table S1 shows predictors used by each prediction model and also gives the numeric variable distributions of their derivation cohorts. Figure 1B illustrates the timeline of the follow-up periods of all models’ derivation cohorts.

### Competence assessment framework for model selection

The key to obtaining an effective ensemble model is a good aggregation mechanism that can choose the best-performing model(s) for individual patients so that an overall optimal classification could be achieved. Stacking methods (learning a model from individual classifiers) usually produce better ensembles than bagging (majority vote or weighted majority vote).^23^ However, the former requires labeled data to further learn a model, which is not possible in our scenario (ie, using the ensemble model in clinical decision making for managing COVID-19). Therefore, a data-independent approach (like bagging) is required. For risk prediction models, their predictive capacities are underpinned by the patient characteristics of their derivation cohorts. For example, given a new patient, models that were trained on (enough number of) similar patients likely perform better than those that were not. The conventional bagging methods (majority vote or their variations) are unlikely to work very well, as they are not capable of capturing such a similarity and its associations with model competence.

We propose a novel bagging mechanism using a competence assessment framework for assisting model selections in the aggregation step. The framework is designed to quantify the competence of each model for a given patient data sample. Three factors are considered. The first factor is called familiarity competence, which quantifies the previously mentioned similarity (ie, how familiar is a model with the new patient sample to be predicted). The second factor is the general competence, which can be reflected by the derivation cohort size, as we know prediction models derived from large cohorts are usually superior to those from smaller ones. The final factor is to consider data completeness of a patient sample relative to a prediction model. “Absolute” data completeness of our validation cohorts is observed to be relatively good, meaning if a clinical feature is collected at a hospital most patients tend to have it. However, “relative” completeness (ie, given a prediction model, the percentage of its risk predictors available in the dataset) varies significantly. Model predictive powers are likely to be compromised by such relative incompleteness, which therefore needs to be considered in the framework.

We first specify the calculation of the familiarity competence. Let $P=\left\{p_{1},\ldots ,p_{k}\right\}$ be the set of all numeric predictors, $\mathrm{dist}\left(m,p\right)=(m_{p},{q1}_{p},{q3}_{p})$ be the distribution (median, first quartile, and third quartile, respectively) of p in the model m’s derivation cohort. Given a patient data sample: $s=\left\{\left(p,v_{p}\right)|p\in P\right\},\;$where $v_{p}$ is the numeric value of predictor p, the familiarity competence of m on p is defined as follows.

$$\begin{array}{c} C_{f}\left(s,m\right)=\sum_{p\in P}\left(1-d\left(v_{p},\mathrm{dist}\left(m,p\right)\right)\right),\mathrm{where}\;\mathrm{is}\;\text{a}\;\text{distance}\;\text{function}\;\text{defined}\;\text{as}\\ d\left(v,\;\left(m,q1,\;q3\right)\right)=\{\begin{array}{cc} 0, & q1\leq v\leq q_{3},\\ \begin{array}{c} \min\left(\frac{\left|v-m\right|}{q3-q1},\;1\right) \end{array} & \text{otherwise} \end{array} \end{array}$$

The final competence calculation is defined as the following formula. The first component divides the familiarity competence by the total number of numeric predictors of the model, incorporating the relative data completeness of s to m. The second component is general competence based on the size of a model’s derivation cohort. Assuming that the 2 components are equally important, we calculate the overall competence as a product of the 2

$$\begin{array}{c} C=\frac{C_{f}\left(s,m\right)}{\left|P_{m}\right|}\times \frac{h\left(m\right)}{\max_{m\in M}h\left(m\right)},\; \end{array}$$

where *P_M_* is the set of all numeric predictors of *m*; *h(m)* is the derivation cohort size and *M* is the set of all models.

### Prediction fusion in ensemble model

Different methods have been proposed in multiple classifier systems^26^ to combine individual classifiers for achieving more accurate classifications. Depending on whether further training is used or not, the combination methods can be categorized as trainable combiners vs nontrainable combiners. The former (eg, AdaBoost)^27^ requires labeled data in the application domain (ie, where the ensemble model is going to be used). The latter (eg, majority vote combiner) can be used in a data-independent manner (ie, applicable in new domains without the need of further training). The motivation of this work is to use the ensemble or combined model to inform decision making in care pathways or policy making, where labeled data are not available. Therefore, nontrainable combiners were used.

A set of fusion methods were implemented. For competence-independent ones, we implemented voting (majority, 1 positive, and 1 negative) and scoring (maximum and average), which are common fusion strategies used in ensemble learning.^26^ When all models are assessed against the data of a given patient, the competence values can then be used to fuse predictions (probabilities of poor prognosis) from all models. We implemented the following: trust-the-most-competent mode (use the prediction of the one with highest competence value); wisdom-of-the-crowd mode (use the weighted average of all predictions); highest-in-top-competent-ones mode (use the maxim probability in top k competent models [k = 3, 5]). Supplementary Figure S2 gives an illustrative example of the 3 fusion strategies. Wisdom of the crowd performed the best in our experiments and was used in this work.

The original model design is another factor that needs to be considered in the prediction fusion. Individual models were designed for predicting different severities: mortality or different definitions of severities. We manually defined a severity score for each model (death models: 1.0; poor prognosis ones: 0.3) and combined those scores in the final fusion formula as follows. The formula considers predictions from all individual models and combines them as weighted average.

$$\begin{array}{c} F\left(s,M\right)=\{\begin{array}{cc} \frac{\sum_{m\in M}\mathrm{Pr}ob\left(m,s\right)\times C\times S_{m}}{\left|M\right|} & \exists m\in M,\;C>0\\ \frac{\sum_{m\in M}\mathrm{Pr}ob\left(m,s\right)\times S_{m}}{\left|M\right|} & \;\text{otherwise} \end{array} \end{array}$$

where *S_m_* is the predefined severity score of *m*.

## RESULTS

**Figure 3.**
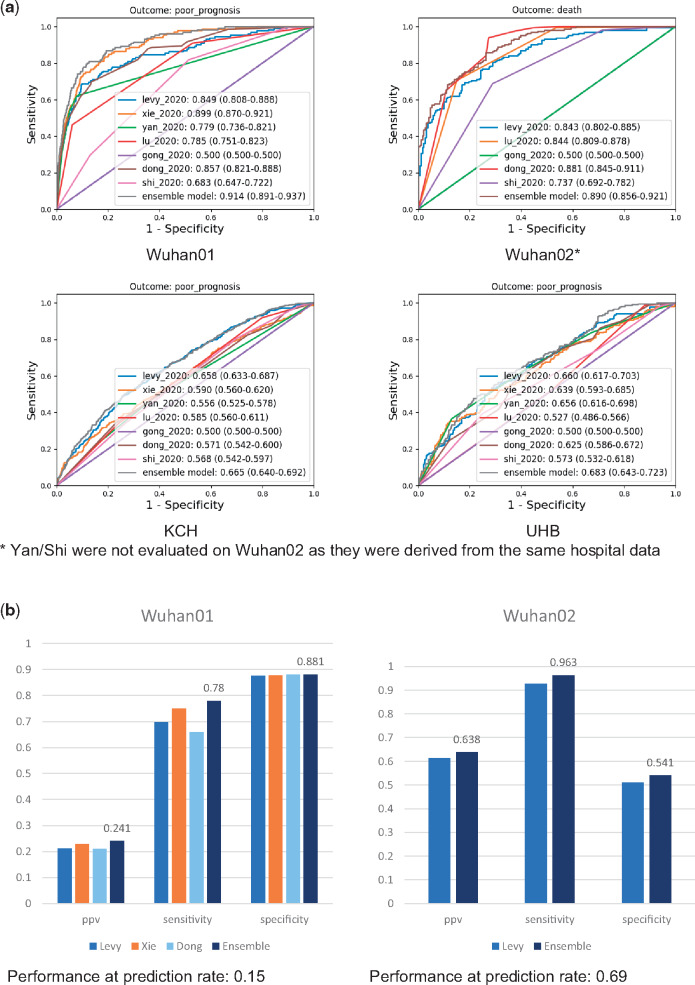

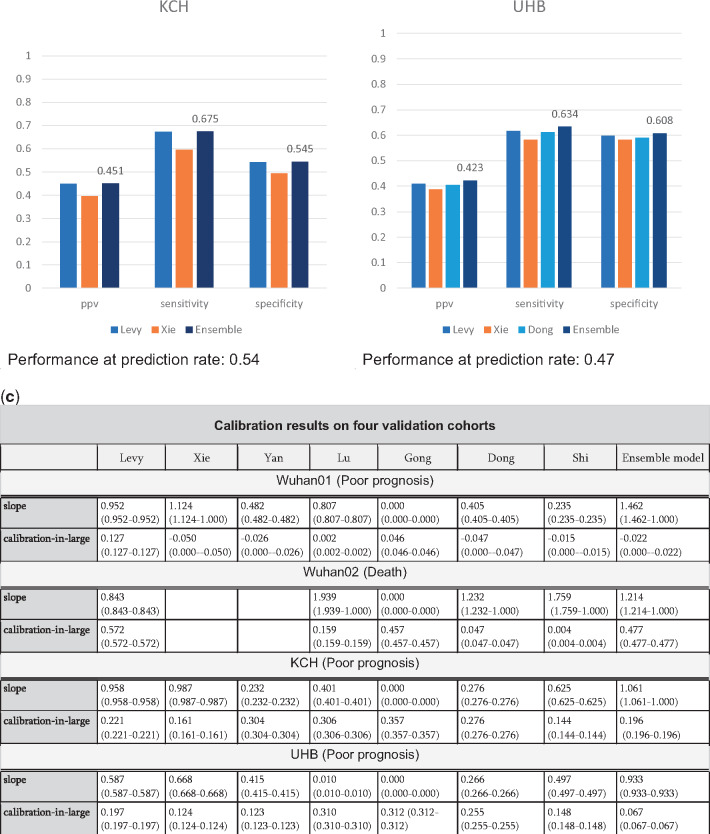
Validation results of discrimination, clinical usefulness, and calibration. **(A)** Discrimination performances: median (95% confidence interval [CI]). **(B)** Positive predictive value (PPV), sensitivity, and specificity of all models validated on cohort-specific prediction rate. Models that could not achieve expected prediction rates were excluded. **(C)** Calibration results on 4 validation cohorts: median (95% CI) where empty cells are for those models that were not validated because they were derived from the same hospital data. KCH: King’s College Hospital; UHB: University Hospitals Birmingham.

The performances of prediction models were evaluated on 3 aspects: discrimination (C-Index), model calibration and a number of parameters defining likely clinical utility. For discrimination (Figure 3A) of individual models, we observed that Xie achieved the best result (C-index, 0.899; 95% confidence interval [CI], 0.874-0.926) on Wuhan01, Dong performed the best (C-index, 0.881; 95% CI, 0.841-0.913) on Wuhan02, and Levy was the best on KCH (C-index, 0.658; 95% CI, 0.629-0.685), and UHB (C-index, 0.660; 95% CI, 0.617-0.713). None of the 7 models examined consistently performed the best across all cohorts, whereas the ensemble model consistently had the best discrimination in all cases: 0.914 (95% CI, 0.891-0.937), 0.890 (95% CI, 0.856-0.921), 0.665 (95% CI, 0.640-0.692), and 0.683 (95% CI, 0.643-0.723) on Wuhan01, Wuhan02, KCH, and UHB respectively. However, the top models (ensemble, Xie, Levy, and Dong) all performed much better on Wuhan cohorts compared with the UK ones. This difference might be explained by the different admission strategies of the 2 countries, indicating that chosen predictors (Figure 1A.3-1A.8) might be less predictive at later stages of clinical presentation and disease progression.

For clinical usefulness, we focus on decision-making support for admission strategies (ie, who to admit and to where). It is not appropriate to use a fixed threshold of probability to validate model performances, as (1) individual models are derived from cohorts with diverse severities and on slightly different definitions of poor prognosis and (2) severity in the validation cohorts also varies significantly. Instead, for each validation cohort we compute an event rate (number of poor prognosis or deceased patients divided by total number of patients) and for models we compute a prediction rate (predicted events divided by total number of patients). We then validate the sensitivity and specificity of a model when its prediction rate is closest to 1.5 times of the event rate or a minimal ratio of 0.15, whichever is larger. Figure 3B shows the performances of all models on 4 cohorts using cohort-specific prediction rate. We observed the ensemble model consistently outperforms individual models across all cohorts on positive predictive value, sensitivity, and specificity. We observed prediction rate–based cutoffs led to quite different performances on the metrics of positive predictive value, sensitivity, and specificity. These were what we expected. For example, for Wuhan01, the mortality rate is 2.4%, which is close to the population level. Therefore, we would expect a good model to have high specificity (ensemble model achieved 0.88) to correctly reject less severe patients so that hospital capacity can be mostly reserved for patients likely to deteriorate (without admitting too many mild patients). On the contrary, when the cohort is very severe (eg, Wuhan02), high sensitivity is preferred (ensemble model: 0.96) as we do not want to discharge those who would likely need intensive care.

To quantify how well the ensemble model reclassifies patients, we also calculated the net reclassification improvements^28^ by comparing them with the best individual model on each validation cohort. Table 3 gives the details, in which the ensemble model achieved net improvements in all cases with the biggest on Wuhan02 and the smallest on KCH.

**Table 3.** Net reclassification improvements of Ensemble model compared with the best individual model on each validation cohort

	Wuhan01 (Ensemble vs Xie)		Wuhan02 (Ensemble vs Dong)		KCH (Ensemble vs Levy)		UHB (Ensemble vs Levy)	
	Event	No Event	Event	No Event	Event	No Event	Event	No Event
Higher	13	132	26	10	51	77	15	42
Lower	7	124	16	17	48	74	11	37
Total	432	2,438	127	230	642	833	325	368
Net reclassification improvements	1.72%		4.83%		0.83%		2.59%	

KCH: King’s College Hospital; UHB: University Hospitals Birmingham.

We also evaluated the model calibrations of all models on all 4 cohorts: Figure 3C shows the calibration slope and calibration in large, and Supplementary Figure S3 depicts the calibration plots. For individual models, similar to C-index performances, they did not perform consistently well across cohorts. For example, Xie had very good calibration on Wuhan01, while it performed poorly on UHB. Again, the ensemble model has shown robust performances on all cohorts—calibrations were good to very good generally.

## DISCUSSION

This work has shown that single models for prediction did not consistently perform well. For example, Dong’s C-index on Wuhan02 is the best in individual models, but it only achieved the fourth-highest C-index on KCH. Similar situations were observed on other top single models, including Xie and Levy. On the one hand, the challenge of getting consistent performances in diverse cohorts resides in the fact that COVID-19 prognosis will vary depending on variables underlying demography (age and comorbidity of the populations) and severities of disease in different settings (because of different admission strategies). For models derived from single cohorts, their prediction capacities were limited by the characteristics of data samples they have seen. Therefore, they are unlikely to achieve a high performance in external cohorts when there are many patients with novel characteristics. On the other hand, ensemble learning methods have the potential to make the best use of all available models. If these models were learned from complementary cohorts, the synergized model will have the potential to achieve better performances than any single model by using most competent ones for individual patients.

Comparing results in the United Kingdom (patients being admitted with more severe disease) and Chinese cohorts (more patients being admitted with mild disease), all models consistently performed worse on UK cohorts. Considering the fact that individual models used quite diverse predictors, adopted different computational algorithms, and were derived from different regions and countries, it seems the observed poorer performances are likely associated with the United Kingdom’s response to the first wave of COVID-19 surge. The United Kingdom mainly admitted severe patients aiming to reserve health service capacities. Therefore, one possible explanation is that blood parameters and physiological measurements are better collected as early as possible to contribute to improved predictive utility.

One limitation of this work was that we were unable to include prediction models that were learned from European cohorts, particularly from the United Kingdom. Including more local models would probably facilitate the ensemble framework to identify those predictors that are more predictive in the European cohorts, which would in turn improve the overall performance in the UK cohorts. In our future work, we will create a web platform to allow the community to share models so that a wide range of diverse and complementary models can be synergized.

## CONCLUSION

In this study we selected and reimplemented 7 prediction models for COVID-19 with diverse derivation cohorts and different implementation techniques. A novel ensemble learning framework was proposed to synergize them for realizing personalized predictions for individual patients. Four international COVID-19 cohorts were used in validating both individual and ensemble models. Validation results showed that ensemble methods could synergize a robust and good-performing model by choosing the most competent model for individual patients.

## FUNDING

HW and HZ are supported by a Medical Research Council and Health Data Research UK Grant (MR/S004149/1), an Industrial Strategy Challenge Grant (MC_PC_18029), and a Wellcome Institutional Translation Partnership Award (PIII054). AK is supported by a Medical Research Council and Health Data Research UK Grant (MR/S003991/1). XW is supported by the National Natural Science Foundation of China (81700006). DMB is funded by a UKRI Innovation Fellowship (Health Data Research UK MR/S00310X/1).TV, FG-S, and TW are funded by National Institute for Health Research (NIHR) covid/non-covid research grants and Queen Elizabeth Hospital Charities. KD is supported by the LifeArc STOPCOVID award. VRC and GVG acknowledge support from the NIHR Birmingham Experimental Cancer Medical Centre, NIHR Birmingham Surgical Reconstruction and Microbiology Research Centre, Nanocommons H2020-EU (731032), and the NIHR Birmingham Biomedical Research Centre and Medical Research Council Health Data Research UK (HDRUK/CFC/01). RJBD is supported by NIHR Biomedical Research Centre at South London and Maudsley NHS Foundation Trust and King’s College London; Health Data Research UK; the BigData@Heart Consortium, funded by the Innovative Medicines Initiative-2 Joint Undertaking under grant agreement no. 116074; the National Institute for Health Research University College London Hospitals Biomedical Research Centre; the UK Research and Innovation London Medical Imaging and Artificial Intelligence Centre for Value Based Healthcare; and the NIHR Applied Research Collaboration South London at King’s College Hospital NHS Foundation Trust.

## AUTHOR CONTRIBUTIONS

HW, HZ, ZI, RD, and BG conceived the study design and developed the study objectives. ZI, HZ, and TS contributed to the statistical analyses. KD provided overall clinical input to the study. HW performed the model reimplementation, ensemble learning, and software development. For King's College Hospital data, DB and JTT were responsible for the data extraction and preparation; JTT, KO, and RZ provided clinical input; and JTT performed data validation. For University Hospitals Birmingham data, AK, VRG, and TV were responsible for data extraction and preparation; FG-S, TW, TV, and GVG provided clinical input and validated the results on University Hospitals Birmingham data. For the Wuhan01 cohort, XW, XZ, XW, and JS extracted the data from the EHR system. HW and HZ preprocessed the raw data and conducted the prediction model validations; BG, HW, HZ, TS, and JS interpreted the data and results. For Wuhan02, Professor Ye Yan and KL were responsible for data extraction and preparation; KL, HW conducted the prediction model validations; YY interpreted the data and results. All authors contributed to the interpretation of the data, critical revision of the manuscript, and approved the final version of the manuscript.

## ETHICS APPROVAL AND CONSENT TO PARTICIPATE

The King's College Hospital component of the project operated under London South East Research Ethics Committee (reference 18/LO/2048) approval granted to the King’s Electronic Records Research Interface (KERRI); specific work on COVID-19 research was reviewed with expert patient input on a virtual committee with Caldicott Guardian oversight. The University Hospitals Birmingham validation was performed as part of a service evaluation agreed with approval from trust research leads and the Caldicott Guardian. The Wuhan validations were approved by the Research Ethics Committee of Shanghai Dongfang Hospital and Taikang Tongji Hospital.

## SUPPLEMENTARY MATERIAL

Supplementary is available at *Journal of the American Medical Informatics Association* online

## Supplementary Material

ocaa295_Supplementary_Data
