# Supplementary material for: Ensemble learning for poor prognosis predictions: A case study on SARS-CoV-2
Source: J Am Med Inform Assoc. 2020 Nov 13;28(4):791–800. doi: 10.1093/jamia/ocaa295 (PMC7717299; doi:10.1093/jamia/ocaa295)
Supplement: ocaa295_Supplementary_Data [file ocaa295_supplementary_data.docx]

Figure S1. COVID-19 poor prognosis model selection process. Literature search was conducted in May 2020.


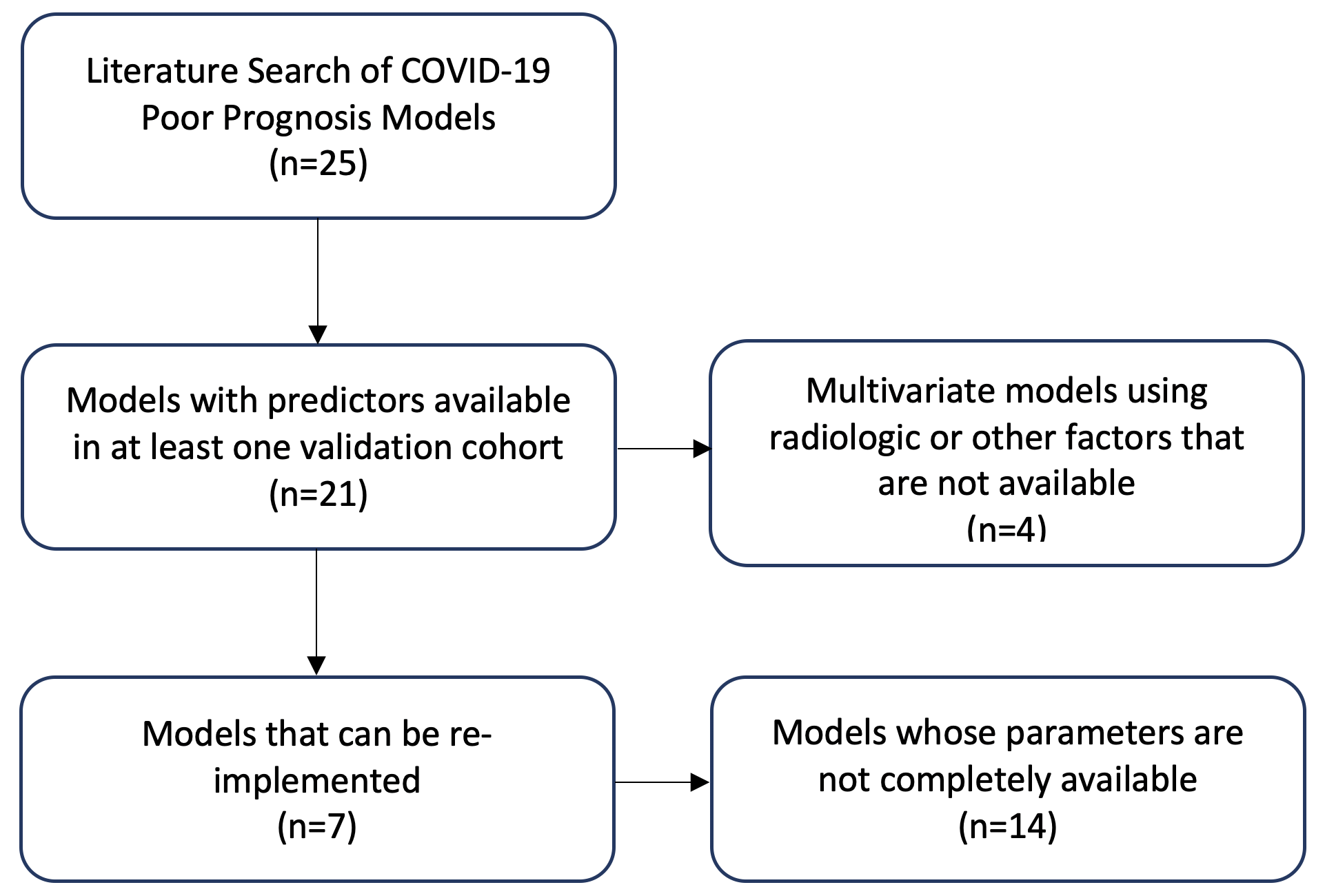


Figure S2. An illustrative example of three fusion strategies: trust-the-most-competent; highest-in-top-competent ones (top 3 in this example); wisdom-of-the-crowd. Each circle denotes a prediction from one model: x-value is the model competence and y-value is the prediction of the model. Red circles denote those selected for prediction fusion computation. The dashed line indicates the fusion result (i.e., the y-value of the line). This work adopted the wisdom-of-the-crowd strategy in the final model.


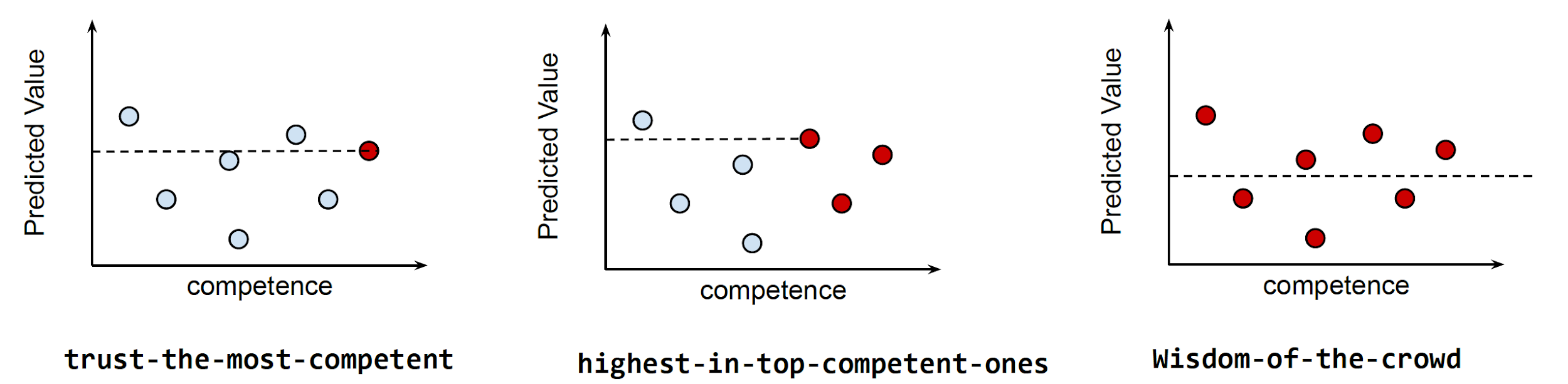


Figure S3. Calibration figures of all models on four validation cohorts. Observed probabilities were for death on Wuhan02 and poor prognosis for others.

| 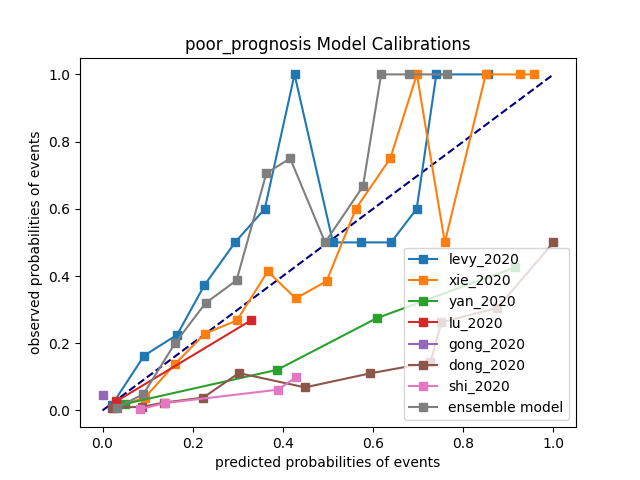  Wuhan01 (outcome: poor prognosis) | 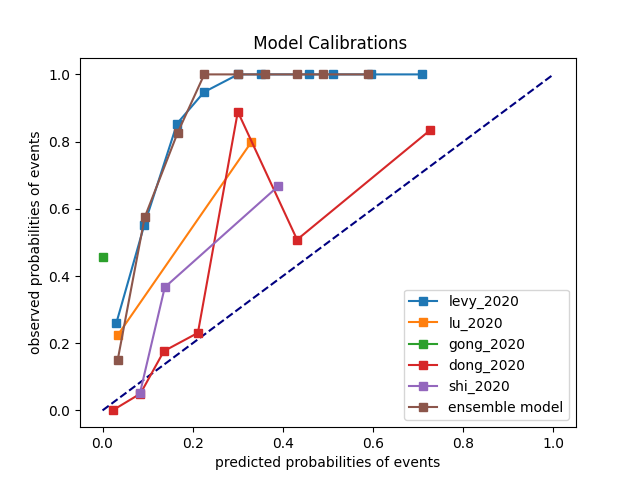  Wuhan 02 (outcome: death) |
| --- | --- |
| 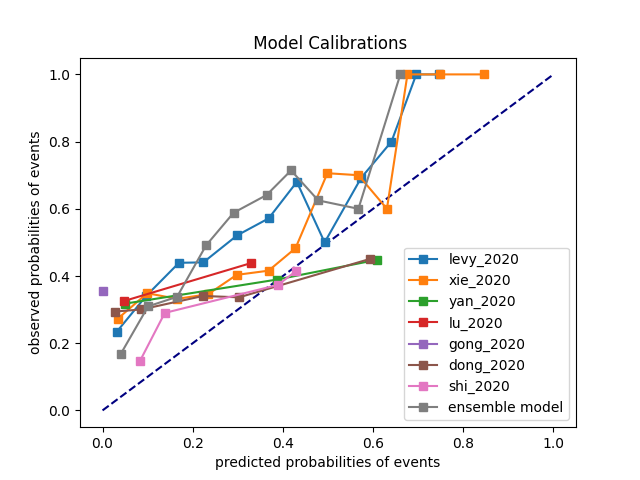  KCH (outcome: poor prognosis) | 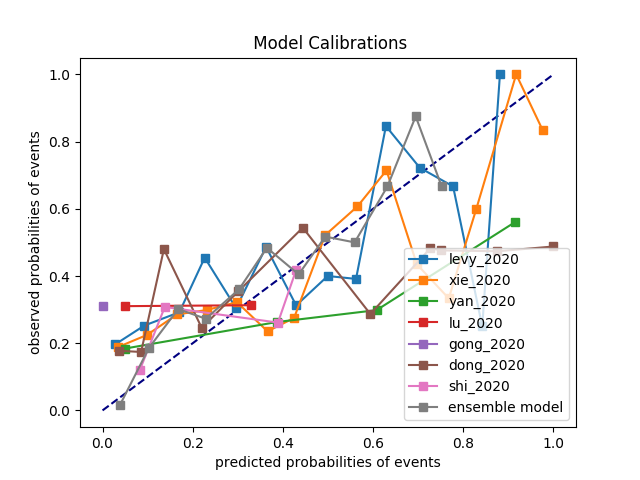  UHB (outcome: poor prognosis) |

|  | **Shi** | **Xie** | **Dong** | **Levy** | **Yan** | **Gong** | **Lu** |
| --- | --- | --- | --- | --- | --- | --- | --- |
| outcome | poor_prognosis | death | poor_prognosis | death | death | poor_prognosis | death |
| model type | scoring | logistic_regression | nomogram | NOCOS | decision_tree | nomogram | scoring |
| **demographics** | | | | | | | |
| Age | 46 [27, 65] | 56.0 [47.8, 67.0] | 40.0 [25.3, 54.7] | 65.0 [54.8, 77.0] | 59 [42, 75] | 45.0 [33, 62.0] | 55 [39, 66] |
| Male | x |  |  |  |  |  |  |
| **underlying conditions** | | | | | | | |
| Comorbidity counts |  |  | x |  |  |  |  |
| Hypertension | x |  |  |  |  |  |  |
| **bloods** | | | | | | | |
| Lactate dehydrogenase |  | 295 [216, 388] | 224 [196, 292] |  | 268.5 [196.0, 365.25] | 175.5 [148.5, 219.5] |  |
| Albumin |  |  |  |  |  | 39.7 [35.1, 44.0] |  |
| C-reactive protein |  |  |  |  | 25.8 [1.98, 98.08] | 5.0 [5.0, 19.5] | 25.7 [8.5, 36.6] |
| Serum sodium |  |  |  | 136.0 [133.0, 139.0] |  |  |  |
| Serum blood urea nitrogen |  |  |  | 18.0 [12.0, 31.0] |  | 3.9 [3.2, 4.6] |  |
| Red cell distribution width |  |  |  | 13.0 [12.8, 14.6] |  | 12.2 [11.8, 12.7] |  |
| Lymphocyte count |  | 0.98 [0.75, 1.52] | 1.4 [0.7, 2.1] |  | 14.35 [4.13, 27.58] |  |  |
| Neutrophil count |  |  |  | 5.82 [4.07, 8.37] |  |  |  |
| Direct bilirubin |  |  |  |  |  | 3.9 [2.7, 5.2] |  |
| **vitals** | | | | | | | |
| Oxygen saturation |  | 97 [95, 98] |  | 96.0 [94.0, 98.0] |  |  |  |

Table S1. Individual prediction model details: outcome, model type, predictors used (x stands for binary predictor used) and numeric predictor value distributions (median [IQR]). Measurements were all collected at admission.

| **Wuhan cohort** | | | | | | |
| --- | --- | --- | --- | --- | --- | --- |
| **Variable** | **N (all)** | **All (n=2869)** | **Did not die (n=2800)** | **Died (n=69)** | **Not poor outcome (n=2738)** | **Poor outcome (n=131)** |
| **Demographic** |  |  |  |  |  |  |
| Age | 2,869 | 60 (50-68) | 60 (49-68) | 71 (64-78) | 60 (49-68) | 70 (63-78) |
| Sex (male) | 2,869 | 1473 (51.3%) | 1425 (50.9%) | 48 (69.6%) | 1389 (50.7%) | 84 (64.1%) |
| Smoking | - | - | - | - | - | - |
| **Comorbidities** |  |  |  |  |  |  |
| Diabetes | 2,869 | 374 (13.0%) | 352 (12.6%) | 22 (31.9%) | 344 (12.6%) | 30 (22.9%) |
| Hypertension | 2,869 | 839 (29.2%) | 802 (28.6%) | 37 (53.6%) | 773 (28.2%) | 66 (50.4%) |
| Heart disease | 2,869 | 248 (8.6%) | 230 (8.2%) | 18 (26.1%) | 219 (8.0%) | 29 (22.1%) |
| Endocrine system diseases | - | - | - | - | - | - |
| Liver disease | - | - | - | - | - | - |
| Metabolic disorders | 2,869 | 90 (3.1%) | 79 (2.8%) | 11 (15.9%) | 75 (2.7%) | 15 (11.5%) |
| Cerebrovascular disease | - | - | - | - | - | - |
| Digestive system diseases | - | - | - | - | - | - |
| Tumor | 2,869 | 110 (3.8%) | 103 (3.7%) | 7 (10.1%) | 101 (3.7%) | 9 (6.9%) |
| COPD | 2,869 | 17 (0.6%) | 15 (0.5%) | 2 (2.9%) | 13 (0.5%) | 4 (3.1%) |
| Other respiratory diseases | 2,869 | 166 (5.8%) | 143 (5.1%) | 23 (33.3%) | 124 (4.5%) | 42 (32.1%) |
| Renal disease | 2,869 | 56 (2.0%) | 48 (1.7%) | 8 (11.6%) | 43 (1.6%) | 13 (9.9%) |
| Urinary system diseases | - | - | - | - | - | - |
| Blood system diseases | - | - | - | - | - | - |
| other_conditions | - | - | - | - | - | - |
| **Admission clinical features** |  |  |  |  |  |  |
| Red cell distribution width | 2,626 | 12.9 (12.4-13.5) | 12.9 (12.4-13.5) | 12.8 (12.3-13.8) | 12.9 (12.3-13.5) | 13.0 (12.5-14.0) |
| Albumin | 2,440 | 38.1 (35.1-40.6) | 38.2 (35.3-40.6) | 31.2 (27.4-34.5) | 38.3 (35.5-40.7) | 31.6 (28.7-35.0) |
| C-reactive protein | 2,432 | 2.3 (0.8-9.0) | 2.2 (0.8-8.2) | 83.0 (28.0-149.2) | 2.1 (0.8-7.3) | 59.9 (14.2-120.0) |
| Serum blood urea nitrogen | 2,474 | 4.4 (3.6-5.5) | 4.3 (3.6-5.4) | 8.6 (5.2-11.9) | 4.3 (3.6-5.4) | 6.8 (5.0-11.0) |
| Lymphocyte count | 2,626 | 1.5 (1.1-1.9) | 1.5 (1.1-1.9) | 0.6 (0.4-0.9) | 1.5 (1.1-1.9) | 0.7 (0.5-1.1) |
| Direct bilirubin | 2,437 | 3.3 (2.5-4.5) | 3.3 (2.5-4.4) | 6.2 (4.5-8.4) | 3.3 (2.5-4.4) | 5.4 (3.5-7.2) |
| Lactate dehydrogenase | 2,341 | 176.8 (151.2-216.0) | 175.5 (150.8-212.4) | 398.2 (331.2-586.3) | 174.6 (150.3-210.2) | 332.2 (244.9-461.0) |
| Serum sodium | 2,383 | 141.6 (139.9-143.2) | 141.6 (140.0-143.2) | 140.8 (136.9-145.1) | 141.6 (140.0-143.2) | 139.8 (137.4-143.4) |
| Neutrophil count | 2,626 | 3.5 (2.7-4.7) | 3.5 (2.7-4.6) | 7.6 (5.3-11.3) | 3.5 (2.7-4.5) | 6.7 (4.8-9.9) |
| **Vital signs** |  |  |  |  |  |  |
| Respiratory rate | 2,865 | 20 (19-21) | 20 (19-21) | 22 (20-24) | 20 (19-21) | 22 (20-24) |
| Systolic blood pressure | 1,452 | 125 (118-134) | 125 (118-133) | 130 (119-140) | 125 (118-133) | 129 (117-138) |
| Diastolic blood pressure | 1,454 | 76 (71-81) | 76 (71-81) | 75 (68-83) | 76 (71-81) | 74 (68-80) |
| Heart rate | 2,866 | 81 (77-86) | 81 (77-86) | 90 (81-98) | 81 (77-85) | 86 (79-95) |
| Temperature | 2,869 | 36 (36-37) | 36 (36-37) | 37 (36-37) | 36 (36-37) | 37 (36-37) |
| Oxygen saturation | 2,851 | 97.8 (97.0-98.2) | 97.8 (97.0-98.2) | 95.2 (92.5-96.6) | 97.8 (97.0-98.2) | 96.6 (94.5-97.7) |

Table S2. Wuhan01 cohort baseline, measurement collected at admission (within 72 hours).

| **KCH cohort** | | | | | | |
| --- | --- | --- | --- | --- | --- | --- |
| **Variable** | **N (all)** | **All (n=1475)** | **Did not die (n=1078)** | **Died (n=397)** | **Not poor outcome (n=949)** | **Poor outcome (n=526)** |
| **Demographic** |  |  |  |  |  |  |
| Age | 1,475 | 71 (57-82) | 66 (54-80) | 80 (70-87) | 69 (54-81) | 75 (60-86) |
| Sex (male) | 1,475 | 844 (57.2%) | 598 (55.5%) | 246 (62.0%) | 514 (54.2%) | 330 (62.7%) |
| Smoking | - | - | - | - | - | - |
| **Comorbidities** |  |  |  |  |  |  |
| Diabetes | 1,475 | 518 (35.1%) | 381 (35.3%) | 137 (34.5%) | 330 (34.8%) | 188 (35.7%) |
| Hypertension | 1,475 | 805 (54.6%) | 570 (52.9%) | 235 (59.2%) | 500 (52.7%) | 305 (58.0%) |
| Heart disease | 1,475 | 290 (19.7%) | 194 (18.0%) | 96 (24.2%) | 176 (18.5%) | 114 (21.7%) |
| Endocrine system diseases | - | - | - | - | - | - |
| Liver disease | - | - | - | - | - | - |
| Metabolic disorders | - | - | - | - | - | - |
| Cerebrovascular disease | - | - | - | - | - | - |
| Digestive system diseases | - | - | - | - | - | - |
| Tumor | - | - | - | - | - | - |
| COPD | 1,475 | 160 (10.8%) | 103 (9.6%) | 57 (14.4%) | 97 (10.2%) | 63 (12.0%) |
| Other respiratory diseases | - | - | - | - | - | - |
| Renal disease | 1,475 | 256 (17.4%) | 154 (14.3%) | 102 (25.7%) | 139 (14.6%) | 117 (22.2%) |
| Urinary system diseases | - | - | - | - | - | - |
| Blood system diseases | - | - | - | - | - | - |
| other_conditions | - | - | - | - | - | - |
| **Admission clinical features** |  |  |  |  |  |  |
| Red cell distribution width | - | - | - | - | - | - |
| Albumin | 1,366 | 37.0 (34.0-40.0) | 38.0 (35.0-41.0) | 35.0 (32.0-39.0) | 38.0 (35.0-41.0) | 36.0 (33.0-39.0) |
| C-reactive protein | 814 | 86.7 (37.0-153.0) | 80.6 (33.1-141.6) | 108.8 (51.9-189.8) | 72.5 (28.8-127.9) | 112.2 (56.8-216.5) |
| Serum blood urea nitrogen | - | - | - | - | - | - |
| Lymphocyte count | 1,380 | 1.0 (0.7-1.4) | 1.0 (0.7-1.4) | 0.9 (0.6-1.4) | 1.0 (0.7-1.4) | 0.9 (0.6-1.4) |
| Direct bilirubin | - | - | - | - | - | - |
| Lactate dehydrogenase | - | - | - | - | - | - |
| Serum sodium | - | - | - | - | - | - |
| Neutrophil count | 1,378 | 5.6 (3.9-8.1) | 5.3 (3.8-7.6) | 6.6 (4.4-9.4) | 5.1 (3.7-7.4) | 6.6 (4.5-9.4) |
| **Vital signs** |  |  |  |  |  |  |
| Respiratory rate | 613 | 20 (18-22) | 19 (18-21) | 22 (20-26) | 19 (18-20) | 23 (20-28) |
| Systolic blood pressure | 592 | 124 (114-133) | 123 (114-133) | 124 (114-139) | 124 (114-132) | 123 (113-137) |
| Diastolic blood pressure | 585 | 71 (65-77) | 72 (66-77) | 70 (62-75) | 71 (66-77) | 70 (63-76) |
| Heart rate | 562 | 84 (76-93) | 83 (76-91) | 87 (79-97) | 82 (75-90) | 88 (80-99) |
| Temperature | 572 | 37 (37-37) | 37 (37-37) | 37 (37-38) | 37 (37-37) | 37 (37-38) |
| Oxygen saturation | 707 | 96.0 (94.6-96.8) | 96.2 (95.3-96.9) | 94.3 (92.1-96.0) | 96.2 (95.4-97.0) | 94.6 (92.6-96.0) |

Table S3. KCH cohort baseline, measurement collected at admission (within 72 hours).

| **Variable** | **N (all)** | **All (n=357)** | **Did not die (n=194)** | **Died (n=163)** | **Not poor outcome (n=194)** | **Poor outcome (n=163)** |
| --- | --- | --- | --- | --- | --- | --- |
| **Demographic** |  |  |  |  |  |  |
| Age | 357 | 62 (46-70) | 51 (37-62) | 69 (62-77) | 51 (37-62) | 69 (62-77) |
| Sex (male) | 357 | 209 (58.5%) | 91 (46.9%) | 118 (72.4%) | 91 (46.9%) | 118 (72.4%) |
| Smoking | - | - | - | - | - | - |
| **Comorbidities** |  |  |  |  |  |  |
| Diabetes | - | - | - | - | - | - |
| Hypertension | - | - | - | - | - | - |
| Heart disease | - | - | - | - | - | - |
| Endocrine system diseases | - | - | - | - | - | - |
| Liver disease | - | - | - | - | - | - |
| Metabolic disorders | - | - | - | - | - | - |
| Cerebrovascular disease | - | - | - | - | - | - |
| Digestive system diseases | - | - | - | - | - | - |
| Tumor | - | - | - | - | - | - |
| COPD | - | - | - | - | - | - |
| Other respiratory diseases | - | - | - | - | - | - |
| Renal disease | - | - | - | - | - | - |
| Urinary system diseases | - | - | - | - | - | - |
| Blood system diseases | - | - | - | - | - | - |
| other_conditions | - | - | - | - | - | - |
| **Admission clinical features** |  |  |  |  |  |  |
| Red cell distribution width | 339 | 12.4 (11.9-13.2) | 12.0 (11.8-12.7) | 12.9 (12.3-13.9) | 12.0 (11.8-12.7) | 12.9 (12.3-13.9) |
| Albumin | 355 | 34.2 (29.9-38.3) | 37.5 (34.2-40.2) | 30.1 (27.6-33.0) | 37.5 (34.2-40.2) | 30.1 (27.6-33.0) |
| C-reactive protein | 344 | 53.3 (12.4-118.3) | 19.5 (3.8-49.8) | 114.1 (61.9-178.8) | 19.5 (3.8-49.8) | 114.1 (61.9-178.8) |
| Serum blood urea nitrogen | - | - | - | - | - | - |
| Lymphocyte count | 355 | 0.8 (0.5-1.2) | 1.1 (0.8-1.5) | 0.6 (0.4-0.8) | 1.1 (0.8-1.5) | 0.6 (0.4-0.8) |
| Direct bilirubin | 355 | 4.5 (2.9-6.7) | 3.5 (2.5-4.7) | 6.2 (4.4-9.2) | 3.5 (2.5-4.7) | 6.2 (4.4-9.2) |
| Lactate dehydrogenase | 355 | 335.0 (238.5-565.0) | 250.0 (202.2-310.5) | 567.0 (427.5-762.0) | 250.0 (202.2-310.5) | 567.0 (427.5-762.0) |
| Serum sodium | 351 | 139.0 (136.2-142.1) | 139.2 (136.5-141.2) | 138.9 (135.8-143.6) | 139.2 (136.5-141.2) | 138.9 (135.8-143.6) |
| Neutrophil count | - | - | - | - | - | - |
| **Vital signs** |  |  |  |  |  |  |
| Respiratory rate | - | - | - | - | - | - |
| Systolic blood pressure | - | - | - | - | - | - |
| Diastolic blood pressure | - | - | - | - | - | - |
| Heart rate | - | - | - | - | - | - |
| Temperature | - | - | - | - | - | - |
| Oxygen saturation | - | - | - | - | - | - |

Table S4. Wuhan02 cohort baseline, measurement collected at admission (within 72 hours).

| **Variable** | **N (all)** | **All (n=693)** | **Did not die (n=561)** | **Died (n=132)** | **Not poor outcome (n=477)** | **Poor outcome (n=216)** |
| --- | --- | --- | --- | --- | --- | --- |
| **Demographic** |  |  |  |  |  |  |
| Age | 693 | 71 (57-81) | 68 (54-79) | 77 (70-83) | 72 (57-82) | 70 (56-80) |
| Sex (male) | 693 | 398 (57.4%) | 320 (57.0%) | 78 (59.1%) | 254 (53.2%) | 144 (66.7%) |
| Smoking | - | - | - | - | - | - |
| **Comorbidities** |  |  |  |  |  |  |
| Diabetes | - | - | - | - | - | - |
| Hypertension | 693 | 367 (53.0%) | 281 (50.1%) | 86 (65.2%) | 240 (50.3%) | 127 (58.8%) |
| Heart disease | - | - | - | - | - | - |
| Endocrine system diseases | - | - | - | - | - | - |
| Liver disease | - | - | - | - | - | - |
| Metabolic disorders | - | - | - | - | - | - |
| Cerebrovascular disease | - | - | - | - | - | - |
| Digestive system diseases | - | - | - | - | - | - |
| Tumor | - | - | - | - | - | - |
| COPD | - | - | - | - | - | - |
| Other respiratory diseases | - | - | - | - | - | - |
| Renal disease | - | - | - | - | - | - |
| Urinary system diseases | - | - | - | - | - | - |
| Blood system diseases | - | - | - | - | - | - |
| other_conditions | - | - | - | - | - | - |
| **Admission clinical features** |  |  |  |  |  |  |
| Red cell distribution width | 687 | 13.8 (12.9-15.3) | 13.7 (12.8-15.1) | 14.2 (13.3-15.9) | 13.7 (12.7-15.4) | 13.9 (13.2-15.1) |
| Albumin | 677 | 30.0 (25.0-34.0) | 30.0 (25.0-34.0) | 29.0 (24.0-32.0) | 31.0 (26.0-35.0) | 28.0 (22.0-32.0) |
| C-reactive protein | 595 | 108.0 (50.5-186.5) | 95.5 (46.8-174.2) | 153.0 (94.0-245.0) | 83.0 (42.0-140.2) | 180.0 (102.5-267.0) |
| Serum blood urea nitrogen | 609 | 6.8 (4.6-11.6) | 6.4 (4.5-10.4) | 9.4 (6.1-14.5) | 6.3 (4.5-10.4) | 8.1 (5.4-13.1) |
| Lymphocyte count | 603 | 0.9 (0.6-1.3) | 0.9 (0.7-1.3) | 0.8 (0.6-1.2) | 0.9 (0.7-1.3) | 0.9 (0.6-1.2) |
| Direct bilirubin | 578 | 10.0 (7.0-15.0) | 10.0 (7.0-15.0) | 11.0 (7.0-18.0) | 10.0 (7.0-14.0) | 11.0 (8.0-20.0) |
| Lactate dehydrogenase | 273 | 370.0 (278.0-504.0) | 362.0 (273.0-488.0) | 417.0 (326.5-588.2) | 316.5 (245.8-411.0) | 436.0 (340.0-623.0) |
| Serum sodium | 688 | 137.0 (135.0-141.0) | 137.0 (135.0-140.0) | 138.0 (135.0-143.0) | 137.0 (134.0-140.0) | 138.0 (135.0-143.0) |
| Neutrophil count | 603 | 5.3 (3.6-7.4) | 5.0 (3.5-7.0) | 6.5 (4.9-9.5) | 4.7 (3.4-6.7) | 6.7 (4.8-9.4) |
| **Vital signs** |  |  |  |  |  |  |
| Respiratory rate | - | - | - | - | - | - |
| Systolic blood pressure | - | - | - | - | - | - |
| Diastolic blood pressure | - | - | - | - | - | - |
| Heart rate | 673 | 87 (76-102) | 86 (74-102) | 92 (78-104) | 86 (74-101) | 92 (78-105) |
| Temperature | 673 | 37 (36-38) | 37 (36-38) | 37 (36-38) | 37 (36-37) | 37 (36-38) |
| Oxygen saturation | 674 | 94.0 (91.0-96.0) | 94.0 (92.0-96.0) | 93.0 (89.0-95.0) | 94.0 (93.0-96.0) | 92.0 (88.0-94.0) |

Table S5. UHB cohort baseline, measurement collected at admission (within 72 hours).
